# Supplementary material for: Characterization and Engineering of Two Novel Strand-Displacing B Family DNA Polymerases from Bacillus Phage SRT01hs and BeachBum
Source: Biomolecules. 2025 Aug 5;15(8):1126. doi: 10.3390/biom15081126 (PMC12383890; doi:10.3390/biom15081126)
Supplement: Supplementary file 1 [file biomolecules-15-01126-s001.zip › Supplementary File S1.pdf]

Polymerase Fidelity Analysis

1 Materials

1.1 Reagents:

Table 1. The information of reagents

Item.No	Name	Brand name
M0494L	Q5® Hot Start High-Fidelity 2X Master Mix	NEB
T1030L	Monarch® RNA Cleanup Kit (5 μg)	NEB
M0262	Lambda Exonuclease	NEB
15630-080	1 M HEPES	Gibco
A600974-0005	TCEP	Sangon Biotech
63069	MagnesiμM Chloride hydrate	Sigma Aldrich
M1787	Manganese (II) chloride Solution	Sigma Aldrich
N0447S	Deoxynucleotide (dNTP) Solution Mix	NEB
60135	3M PotassiμM chloride solution	Sigma Aldrich
AM9937	Nuclease-Free Water	Thermo Scientific™
M0202M	T4 DNA Ligase	NEB
B4252	X-Gal	Sigma Aldrich
ER1751	PfoI (10 U/μL)	Thermo Scientific
ER1932	LguI (SapI) (5 U/μL)	Thermo Scientific
DP209-03	TIANgel Midi Purification Kil	TIANGEN
10164ES08	2× Hieff Canace® AdvanceFast PCR Master Mix	Yeasen

1.2 Plasmid and primer sequences

Y3: 5’Pho-TGACACATGCAGCTCCCGGAGAC-3’;

Y4: 5’-AGGAAGCGGAAGAGCGCCCAATA-3’;

Y34 PCR fragment:
AGGAAGCGGAAGAGCGCCCAATACGCAAACCGCCTCTCCCCGCGCGTTGGCCGA
TTCATTAATGCAGCTGGCACGACAGGTTTCCCGACTGGAAAGCGGGCAGTGAGC
GCAACGCAATTAATGTGAGTTAGCTCACTCATTAGGCACCCCAGGCTTTACACTT
TATGCTTCCGGCTCGTATGTTGTGTGGAATTGTGAGCGGATAACAATTTCACACA
GGCCTCAGCTATGACCATGATTACGAATTCGAGCTCGGTACCCGGGGATCCTCTA
GAGTCGACCTGCAGGCATGCAAGCTTGGCACTGGCCGTCGTTTTACAACGTCGTG
ACTGGGAAAACCCTGGCGTTACCCAACTTAATCGCCTTGCAGCACATCCCCCTTT
CGCCAGCTGGCGTAATAGCGAAGAGGCCCGCACCGATCGCCCTTCCCAACAGTTG
CGCAGCCTGAATGGCGAATGGCGCCTGATGCGGTATTTTCTCCTTACGCATCTGT
GCGGTATTTCACACCGCATATGGTGCACTCTCAGTACAATCTGCTCTGATGCCGC
ATAGTTAAGCCTGAGCCGACACCCGCCAACACCCGCTGACGCGCCCTGACGGGCT
TGTCTGCTCCCGGCATCCGCTTACAGACAAGCTGTGACCGTCTCCGGGAGCTGCA
TGTGTCA

PUC18_pSJI:
TCGCGCGTTTCGGTGATGACGGTGAAAACCTCTGACACATGCAGCTCCCGGAGAC
GGTCACAGCTTGTCTGTAAGCGGATGCCGGGAGCAGACAAGCCCGTCAGGGCGC
GTCAGCGGGTGTTGGCGGGTGTCGGCTCAGGCTTAACTATGCGGCATCAGAGCAG
ATTGTACTGAGAGTGCACCATATGCGGTGTGAAATACCGCACAGATGCGTAAGGAG
AAAATACCGCATCAGGCGCCATTCGCCATTCAGGCTGCGCAACTGTTGGGAAGGG
CGATCGGTGCGGGCCTCTTCGCTATTACGCCAGCTGGCGAAAGGGGGATGTGCTG
CAAGGCGATTAAGTTGGGTAACGCCAGGGTTTTCCCAGTCACGACGTTGTAAAAC
GACGGCCAGTGCCAAGCTTGCATGCCTGCAGGTCGACTCTAGAGGATCCCCGGGT
ACCGAGCTCGAATTCGTAATCATGGTCATAGCTGAGGCCTGTGTGAAATTGTTATCC
GCTCACAATTCCACACAACATACGAGCCGGAAGCATAAAGTGTAAAGCCTGGGGT
GCCTAATGAGTGAGCTAACTCACATTAATTGCGTTGCGCTCACTGCCCGCTTTCCA
GTCGGGAAACCTGTCGTGCCAGCTGCATTAATGAATCGGCCAACGCGCGGGGAGA
GGCGGTTTGCGTATTGGGCGCTCTTCCGCTTCCTCGCTCACTGACTCGCTGCGCTC
GGTCGTTCGGCTGCGGCGAGCGGTATCAGCTCACTCAAAGGCGGTAATACGGTTAT
CCACAGAATCAGGGGATAACGCAGGAAAGAACATGTGAGCAAAAGGCCAGCAAA
AGGCCAGGAACCGTAAAAAGGCCGCGTTGCTGGCGTTTTTCCATAGGCTCCGCCC
CCCTGACGAGCATCACAAAAATCGACGCTCAAGTCAGAGGTGGCGAAACCCGAC
AGGACTATAAAGATACCAGGCGTTTCCCCCTGGAAGCTCCCTCGTGCGCTCTCCTG
TTCCGACCCTGCCGCTTACCGGATACCTGTCCGCCTTTCTCCCTTCGGGAAGCGTG
GCGCTTTCTCATAGCTCACGCTGTAGGTATCTCAGTTCGGTGTAGGTCGTTCGCTCC
AAGCTGGGCTGTGTGCACGAACCCCCCGTTCAGCCCGACCGCTGCGCCTTATCCG
GTAACTATCGTCTTGAGTCCAACCCGGTAAGACACGACTTATCGCCACTGGCAGCA
GCCACTGGTAACAGGATTAGCAGAGCGAGGTATGTAGGCGGTGCTACAGAGTTCT
TGAAGTGGTGGCCTAACTACGGCTACACTAGAAGGACAGTATTTGGTATCTGCGCT
CTGCTGAAGCCAGTTACCTTCGGAAAAAGAGTTGGTAGCTCTTGATCCGGCAAAC
AAACCACCGCTGGTAGCGGTGGTTTTTTTGTTTGCAAGCAGCAGATTACGCGCAG
AAAAAAAGGATCTCAAGAAGATCCTTTGATCTTTTCTACGGGGTCTGACGCTCAGT
GGAACGAAAACTCACGTTAAGGGATTTTGGTCATGAGATTATCAAAAAGGATCTTC
ACCTAGATCCTTTTAAATTAAAAATGAAGTTTTAAATCAATCTAAAGTATATATGAGT
AAACTTGGTCTGACAGTTACCAATGCTTAATCAGTGAGGCACCTATCTCAGCGATC
TGTCTATTTCGTTCATCCATAGTTGCCTGACTCCCCGTCGTGTAGATAACTACGATAC
GGGAGGGCTTACCATCTGGCCCCAGTGCTGCAATGATACCGCGAGACCCACGCTC
ACCGGCTCCAGATTTATCAGCAATAAACCAGCCAGCCGGAAGGGCCGAGCGCAGA
AGTGGTCCTGCAACTTTATCCGCCTCCATCCAGTCTATTAATTGTTGCCGGGAAGCT
AGAGTAAGTAGTTCGCCAGTTAATAGTTTGCGCAACGTTGTTGCCATTGCTACAGG
CATCGTGGTGTCACGCTCGTCGTTTGGTATGGCTTCATTCAGCTCCGGTTCCCAAC
GATCAAGGCGAGTTACATGATCCCCCATGTTGTGCAAAAAAGCGGTTAGCTCCTTC
GGTCCTCCGATCGTTGTCAGAAGTAAGTTGGCCGCAGTGTTATCACTCATGGTTAT
GGCAGCACTGCATAATTCTCTTACTGTCATGCCATCCGTAAGATGCTTTTCTGTGAC
TGGTGAGTACTCAACCAAGTCATTCTGAGAATAGTGTATGCGGCGACCGAGTTGCT
CTTGCCCGGCGTCAATACGGGATAATACCGCGCCACATAGCAGAACTTTAAAAGTG
CTCATCATTGGAAAACGTTCTTCGGGGCGAAAACTCTCAAGGATCTTACCGCTGTT
GAGATCCAGTTCGATGTAACCCACTCGTGCACCCAACTGATCTTCAGCATCTTTTA

CTTTCACCAGCGTTTCTGGGTGAGCAAAAACAGGAAGGCAAAATGCCGCAAAAA AGGGAATAAGGGCGACACGGAAATGTTGAATACTCATACTCTTCCTTTTTCAATATT ATTGAAGCATTTATCAGGGTTATTGTCTCATGAGCGGATACATATTTGAATGTATTTA GAAAAATAAACAAATAGGGGTTCCGCGCACATTTCCCCGAAAAGTGCCACCTGAC GTCTAAGAAACCATTATTATCATGACATTAACCTATAAAAATAGGCGTATCACGAGG CCCTTTCGTC

2 Template construction

A. PCR was performed using Q5® Hot Start High-Fidelity 2X Master Mix with Y3 and Y4 as primers (with EcoO109I and PciI cleavage sites, respectively) and PUC18_pSJI plasmids as templates.

Table 2. PCR system for template amplification

Reagent	Original concentration	Final concentration	Volume (μL)
2X Q5 Mix	2 X	1 X	25
0.5 μM Y3	100 μM	0.5 μM	0.25
0.5 μM Y4	100 μM	0.5 μM	0.25
Template-PUC18_pSJI	300 ng	6 ng/μL	
ddH2O			To 50

Table 3. The process of PCR system

PCR reaction temperature conditions		
	98℃	30 s
	98℃	10 s
30cycles	60℃	30 s
	72℃	10 s
	72℃	2 min

B. The target fragment (665bp) containing the LacZα region was obtained using NEB PCR Clean-up kit.

C. The PCR product was then single-stranded digested with lambda exonuclease to obtain single-stranded DNA at 37°C for 60min and then 75°C for 10min.

Table 4. The reaction system and reaction conditions of digestion.

Reagent	Original concentration	Final concentration	Volume (μL)
10X lambda Buffer	10 X	1 X	5
Lambda exonuclease	5 U/μL	0.2 U/μL	2
Template	200 ng/μL	20 ng/μL	5
ddH2O			To 50

D. 100 μM of a primer (Y3) with a 5' phosphate group (Y3) was annealing to 50 μM of single-stranded Y34 at 60°C for 5min.

3 PCR amplification using the above template complex and polymerase mutants.

PCR was performed at 30°C for 60min. The target fragments were purified by the NEB PCR Clean-up kit,

Table 5. The PCR reaction system.

Reagent	Original concentration	Final concentration	Volume (μL)
HEPES pH7.5	1 M	20 mM	0.4
TCEP	0.5 M	4 mM	0.16
MgCl2/MnCl2	50 mM	1 mM	0.4
dNTPs	10 mM	100 μM	0.2
KCl	3 M	150 mM	1
Template	10 ng/μL	5 ng/μL	10
Polymerase		100 nM	
ddH2O			To 20 μL

4 Ligation the target fragments with plasmids for blue-white selection.

A. The above target fragments were digested by PfoI and SapI and recycled by the NEB PCR Clean-up kit.

B. The PUC18_pSJI plasmids were also digested with PfoI and SapI and recovered by TIANgel Midi purification kit (Tiangen Biotech).

C. The ligation was performed by T4 ligase at 25°C for 30min. The ratio of the target fragments and plasmids was 6:1 in the reaction system.

D. The ligation products were transformed to the Top10 competent cells.

E. Blue and white selection

40 μL of 40 mg/mL X-Gal and 4 μL of 1 M IPTG were mixed and incubated on LB solid medium containing 100 μg/mL ampicillin antibiotic. 75 μL of *E. coli* was incubated on the above solid medium at 37°C for 16~18 hours.

F. The number of blue and white single colonies was counted using ImageJ software and the percentage of blue and white single colonies was calculated.

G. Test the proportion of while colonies with target fragments among all while colonies.

White single colonies were selected and cultured. The PCR was performed using primers Y3 and Y4.

Table 6. PCR amplification system

Reagent	Original concentration	Final concentration	Volume (μL)
2 X Hieff Mix	2X	1X	5
Y3	100μM	0.5μM	0.05
Y4	100μM	0.5μM	0.05
Bacterial			1
ddH_2O			To 10

The PCR results were analyzed by a 1% agarose gel.

H. Calculation of mutation rates

We will normalize the number and percentage of white colonies and calculate the error rate using the equation = [white colonies / (white colonies + blue colonies)]/ fragments length.

I. Statistical results

Table 7. The results of blue and white colonies for BBum_Pro_L and Phi29 polymerases

Sample Name/Catalytic ions	White colonies + Blue colonies	Blue colonies	White colonies
BBum_Pro_L_replicate 1 (Mg^{2+})	1927	1926	1
BBum_Pro_L_replicate 2 (Mg^{2+})	2437	2437	0
BBum_Pro_L_replicate 3 (Mg^{2+})	1976	1974	2
BBum_Pro_L_replicate 4 (Mg^{2+})	2558	2557	1
BBum_Pro_L_replicate 5 (Mg^{2+})	2015	2015	0
BBum_Pro_L_replicate 6 (Mg^{2+})	1835	1835	0
BBum_Pro_L_replicate 7 (Mg^{2+})	2032	2031	1
BBum_Pro_L_replicate 8 (Mg^{2+})	2163	2162	1
Phi29_replicate 1 (Mg^{2+})	2471	2470	1
Phi29_replicate 2 (Mg^{2+})	2600	2599	1
Phi29_replicate 3 (Mg^{2+})	2459	2459	0
Phi29_replicate 4 (Mg^{2+})	2846	2846	0
Phi29_replicate 5 (Mg^{2+})	2070	2070	0
Phi29_replicate 6 (Mg^{2+})	2258	2258	0
Phi29_replicate 7 (Mg^{2+})	2055	2054	1
Phi29_replicate 8 (Mg^{2+})	2491	2490	1
Phi29_replicate 1 (Mn^{2+})	2338	2334	4
Phi29_replicate 2 (Mn^{2+})	2209	2204	5
Phi29_replicate 3 (Mn^{2+})	1920	1918	2
Phi29_replicate 4 (Mn^{2+})	2557	2550	7
Phi29_replicate 5 (Mn^{2+})	2401	2392	9

Phi29_replicate 6	(Mn^{2+})	1106	1102	4
Phi29_replicate 7	(Mn^{2+})	1021	1015	6
Phi29_replicate 8	(Mn^{2+})	1051	1048	3

Based on the above results (Table 7), we obtained the mean value across all replicates and calculated fidelity (Table 8).

Table 8. The Fidelity of BBum_Pro_L and Phi29 polymerases

Sample Name/Catalytic ions	Mean (White colonies + Blue colonies)	Mean (Blue colonies)	Mean (White colonies)	Fidelity
BBum_Pro_L (Mg^{2+})	2117.875	2117.125	0.75	5.32524E-07
Phi29 (Mg^{2+})	2406.25	2405.75	0.5	3.12469E-07
Phi29 (Mn^{2+})	1825.375	1820.375	5	4.11904E-06
