# Supplementary material for: Characterization and Engineering of Two Novel Strand-Displacing B Family DNA Polymerases from Bacillus Phage SRT01hs and BeachBum
Source: Biomolecules. 2025 Aug 5;15(8):1126. doi: 10.3390/biom15081126 (PMC12383890; doi:10.3390/biom15081126)
Supplement: Supplementary file 1 [file biomolecules-15-01126-s001.zip › Supplementary File S2.pdf]

Supplementary File S2：All the original gel images

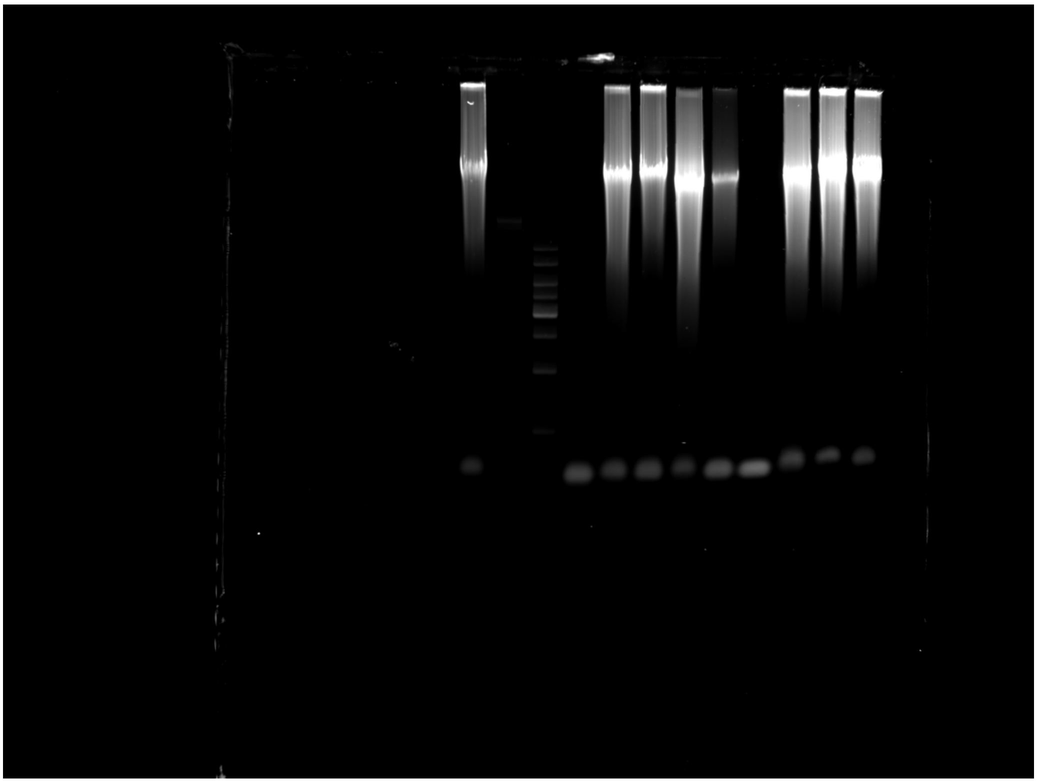

Figure 1C

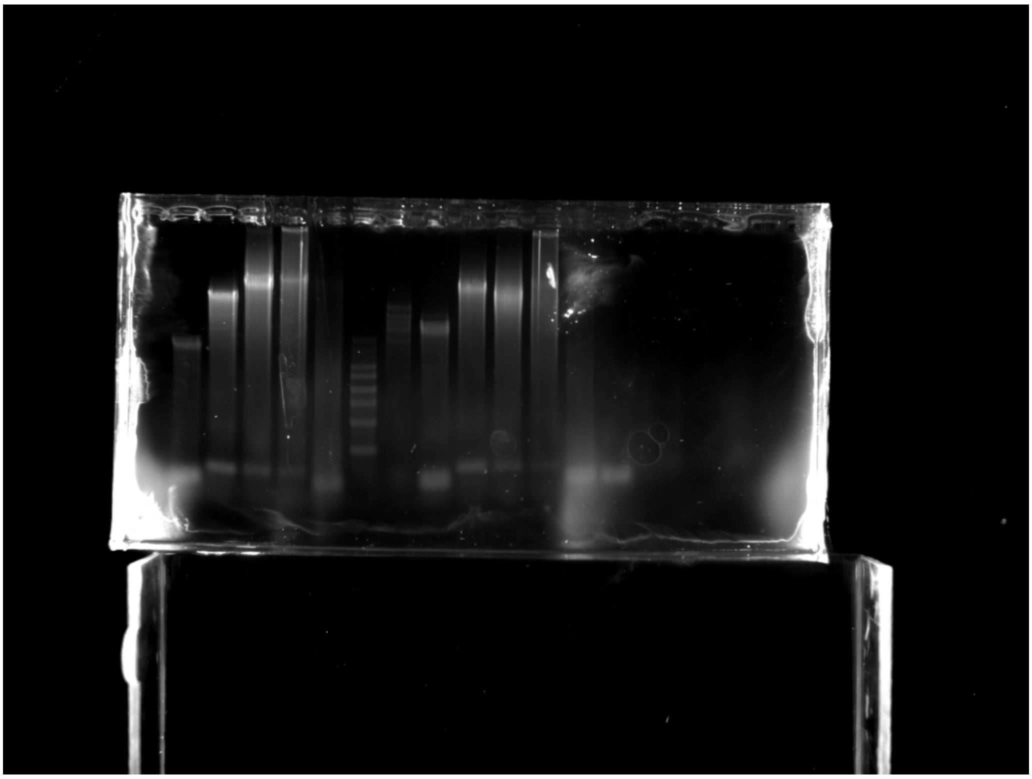

Figure 2A

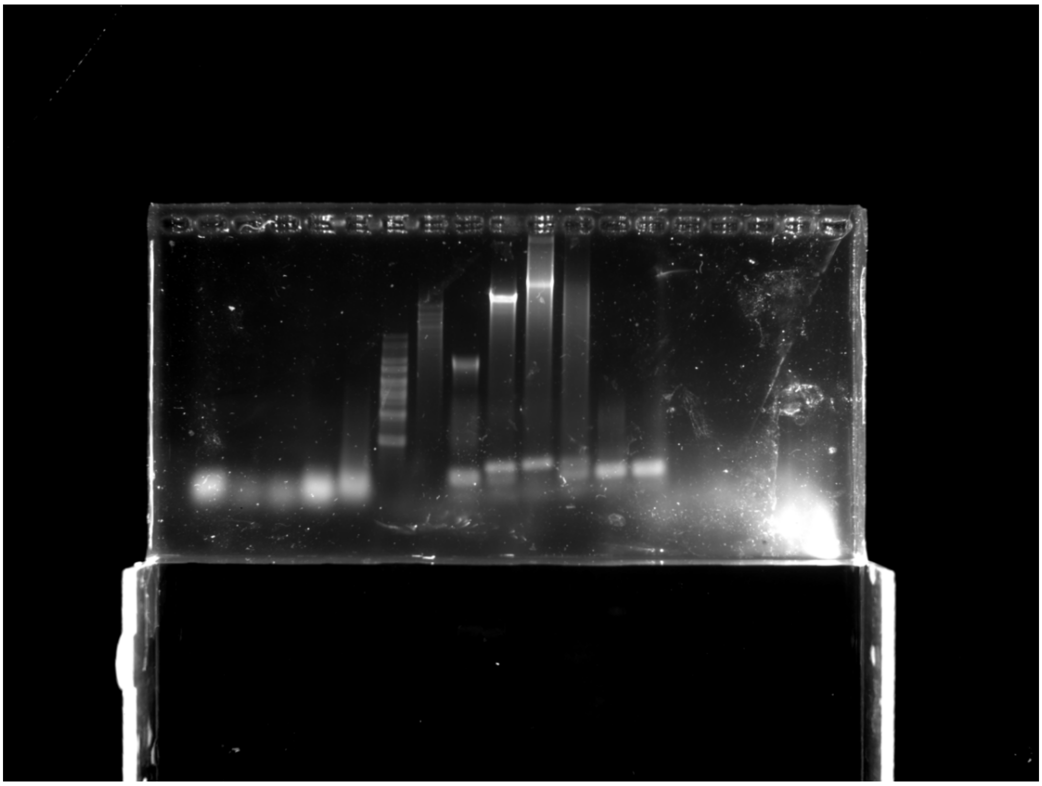

Figure 2B

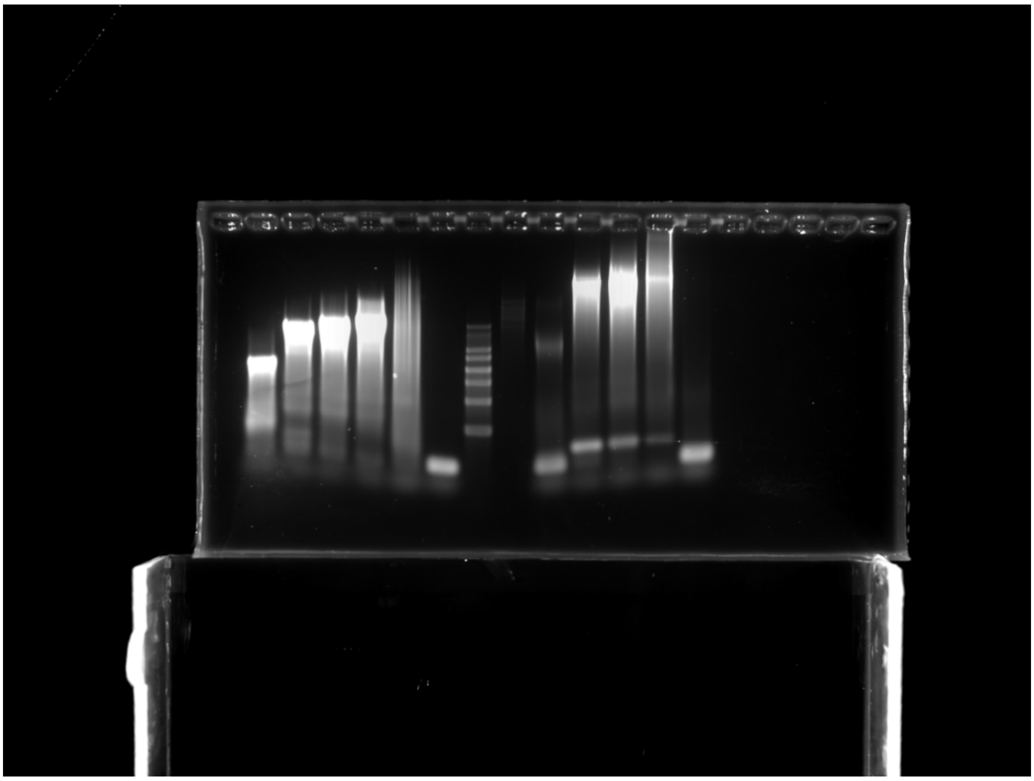

Figure 2C

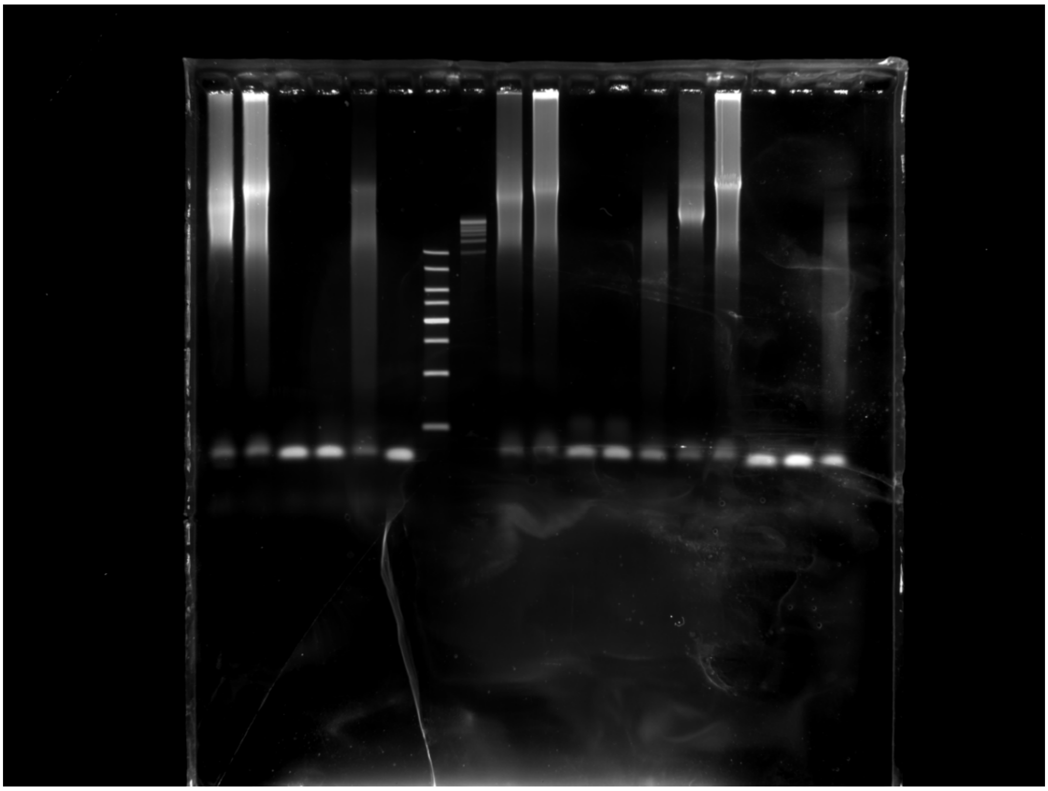

Figure 2D

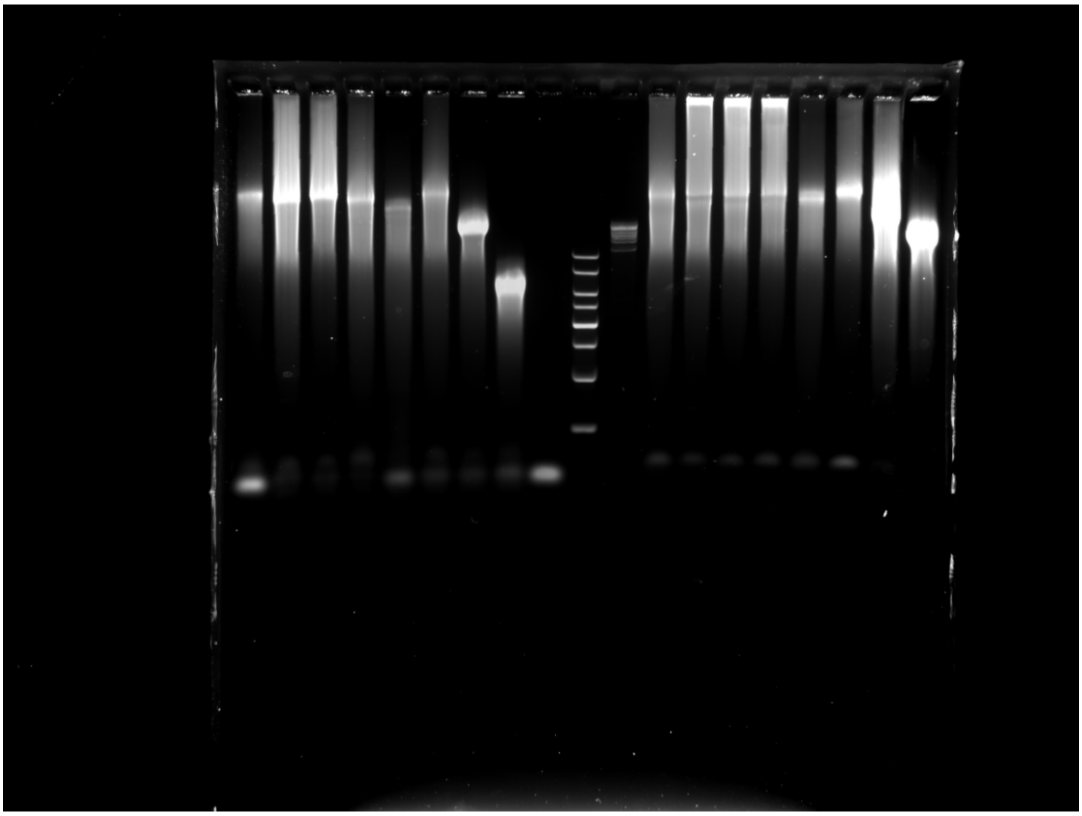

Figure 5A

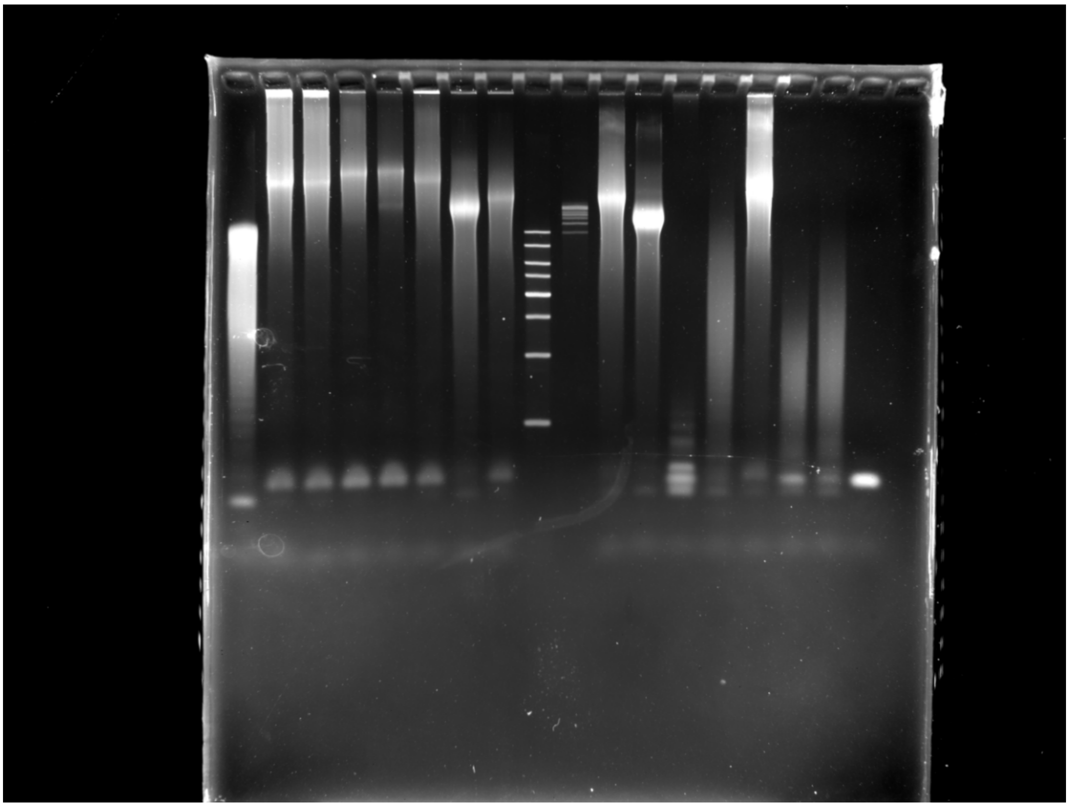

Figure 5B

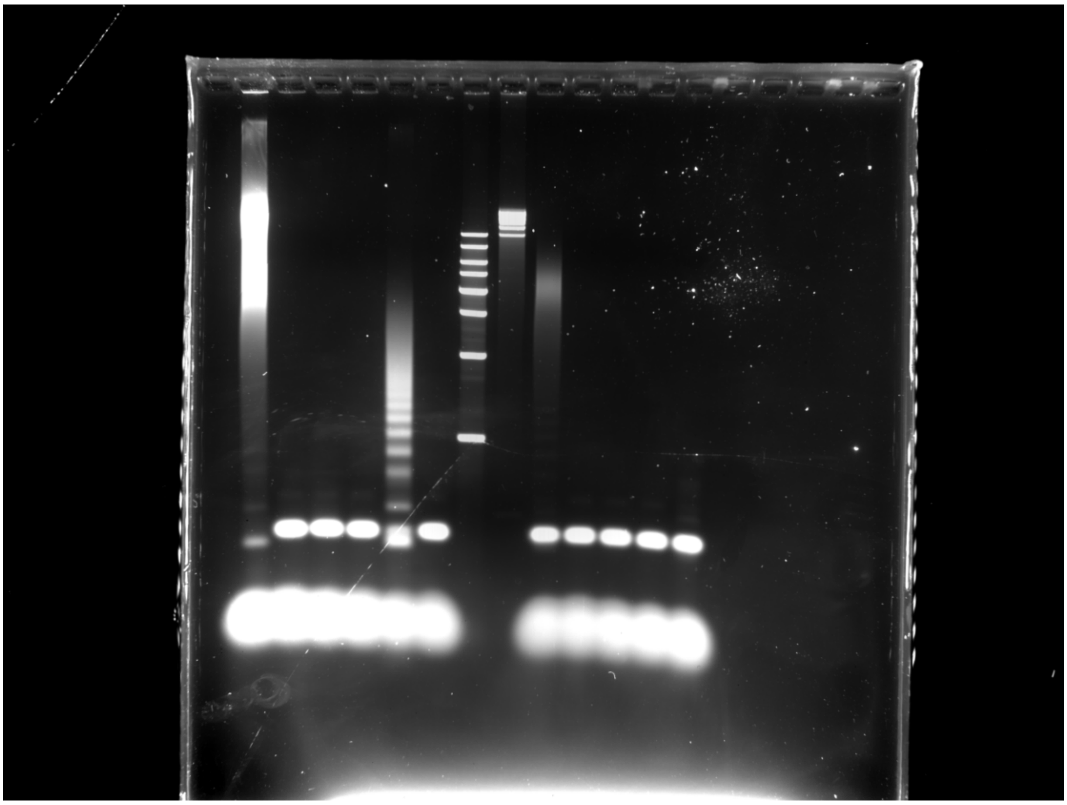

Figure 6A

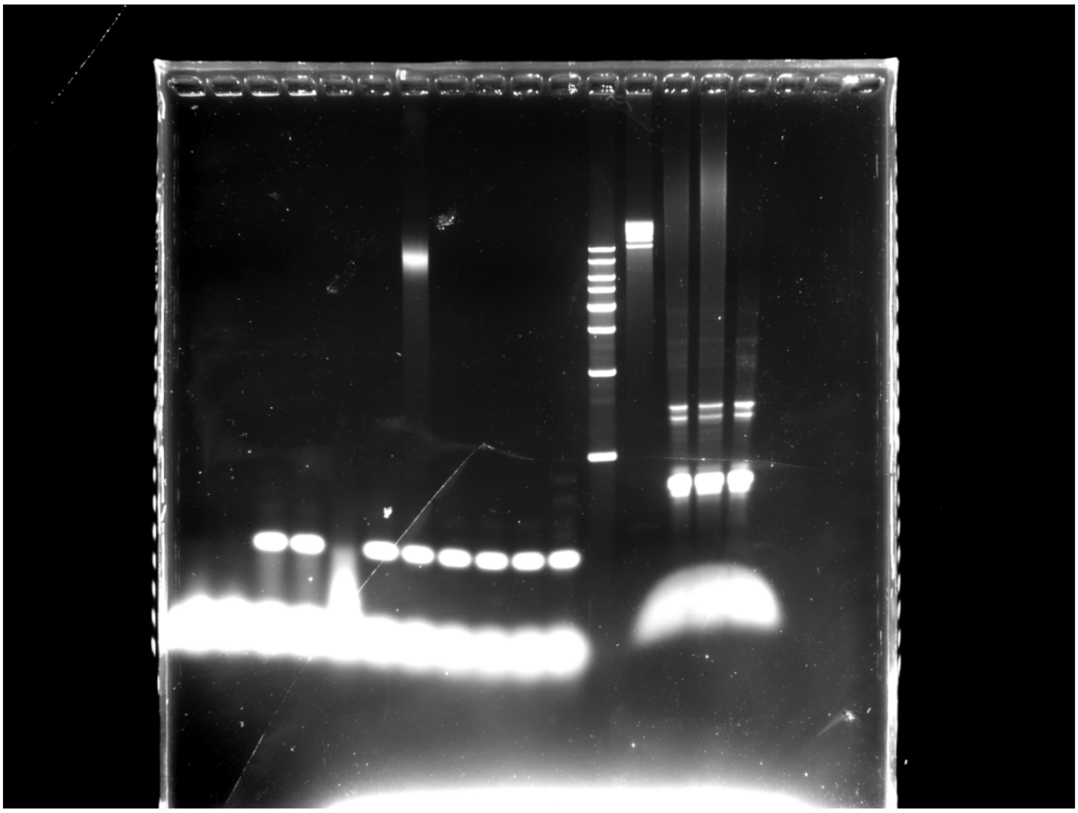

Figure 6B

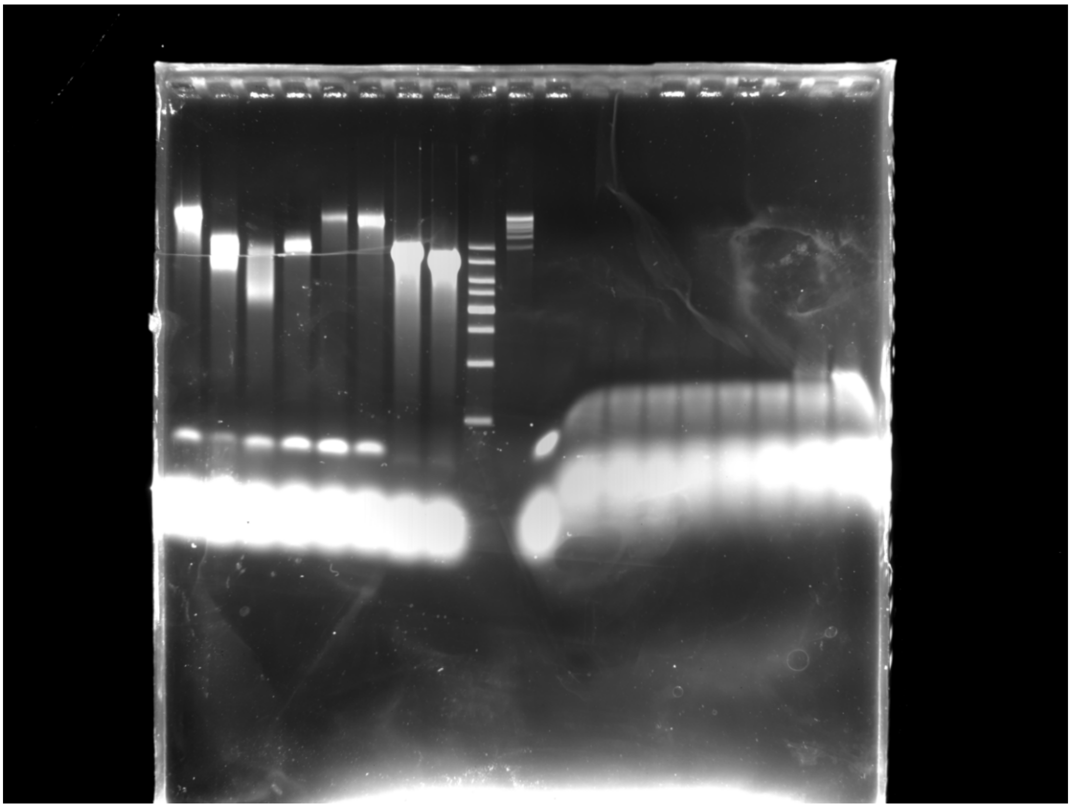

Figure 6C

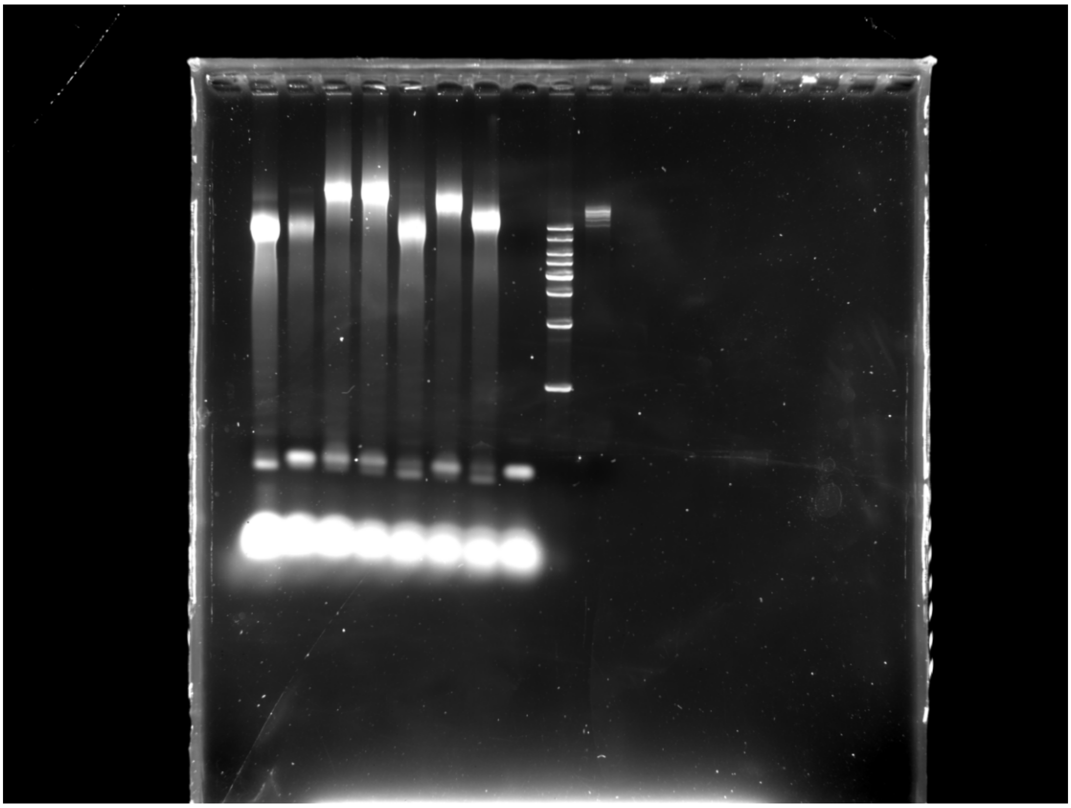

Figure 6D

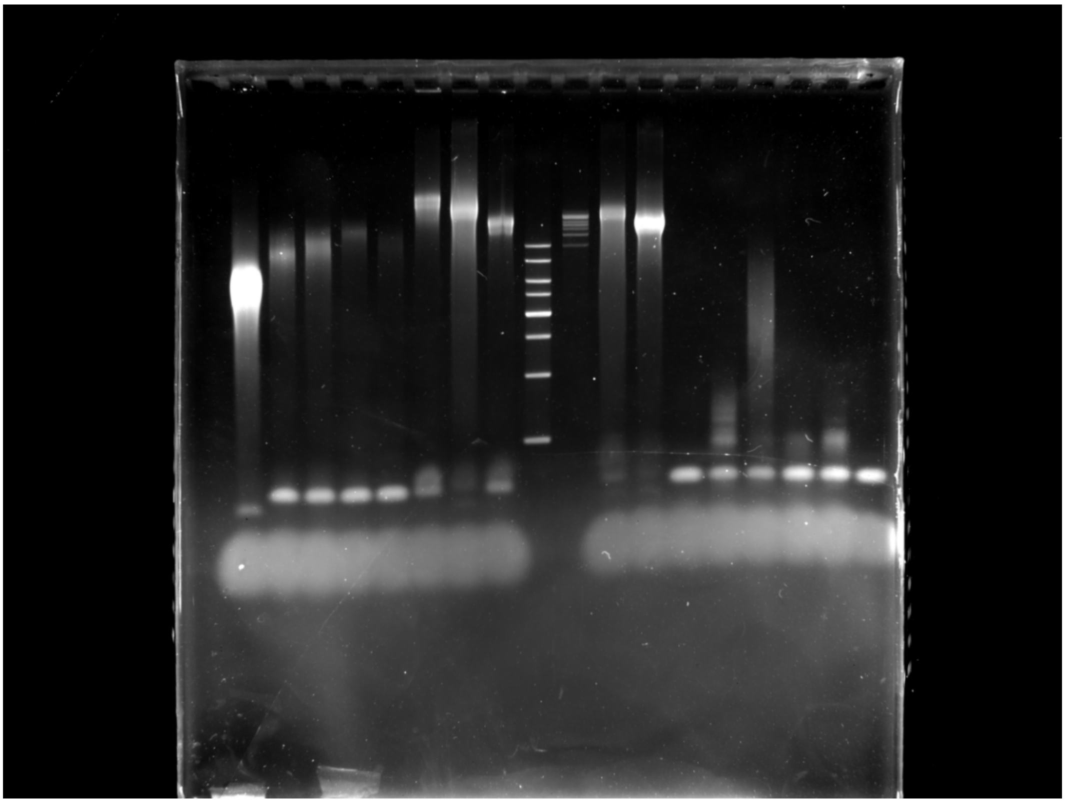

Figure 6E

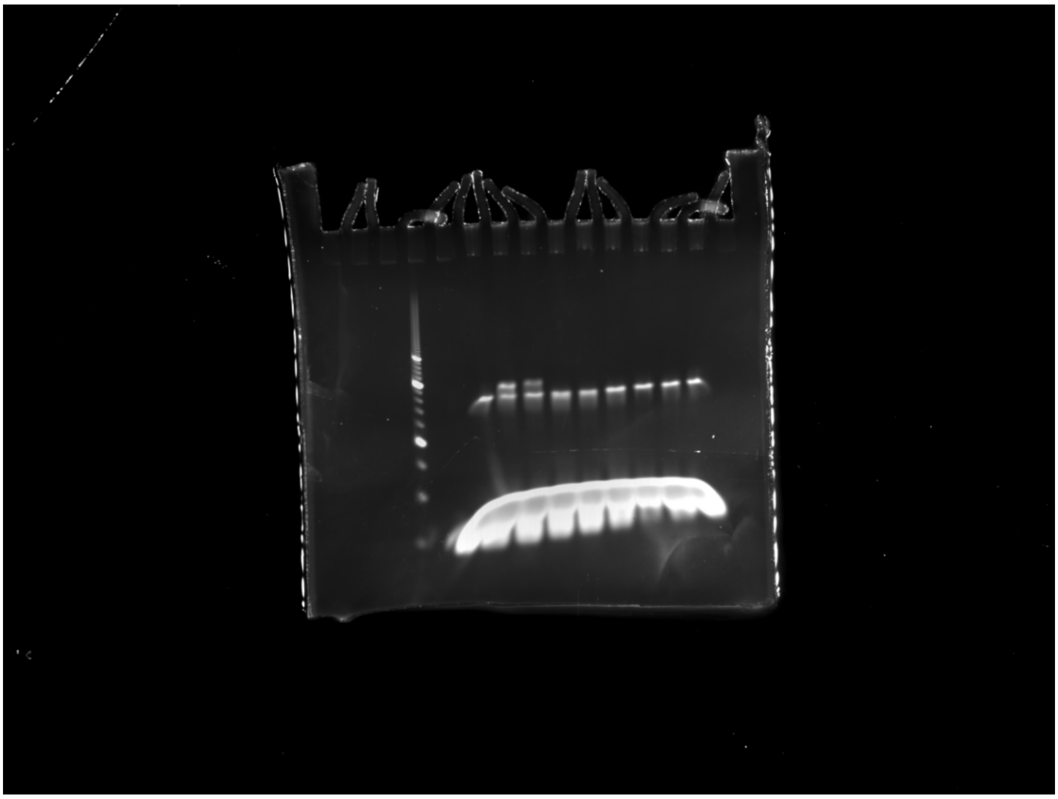

Figure 7A

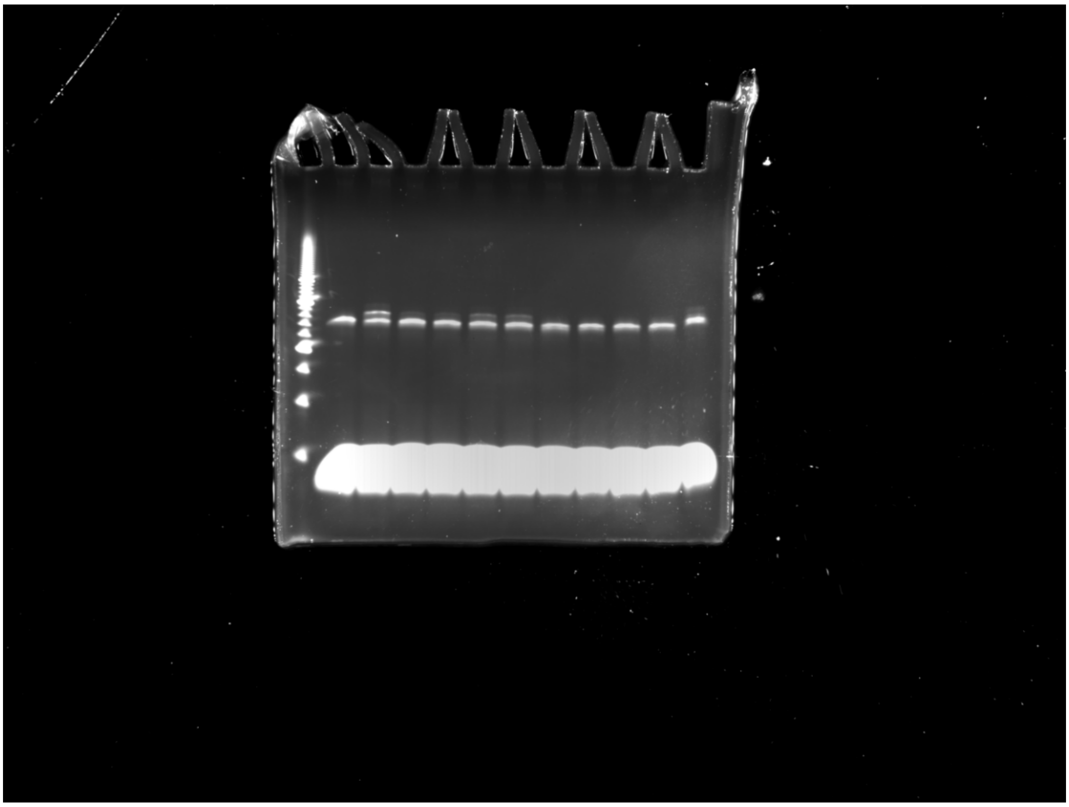

Figure 7B

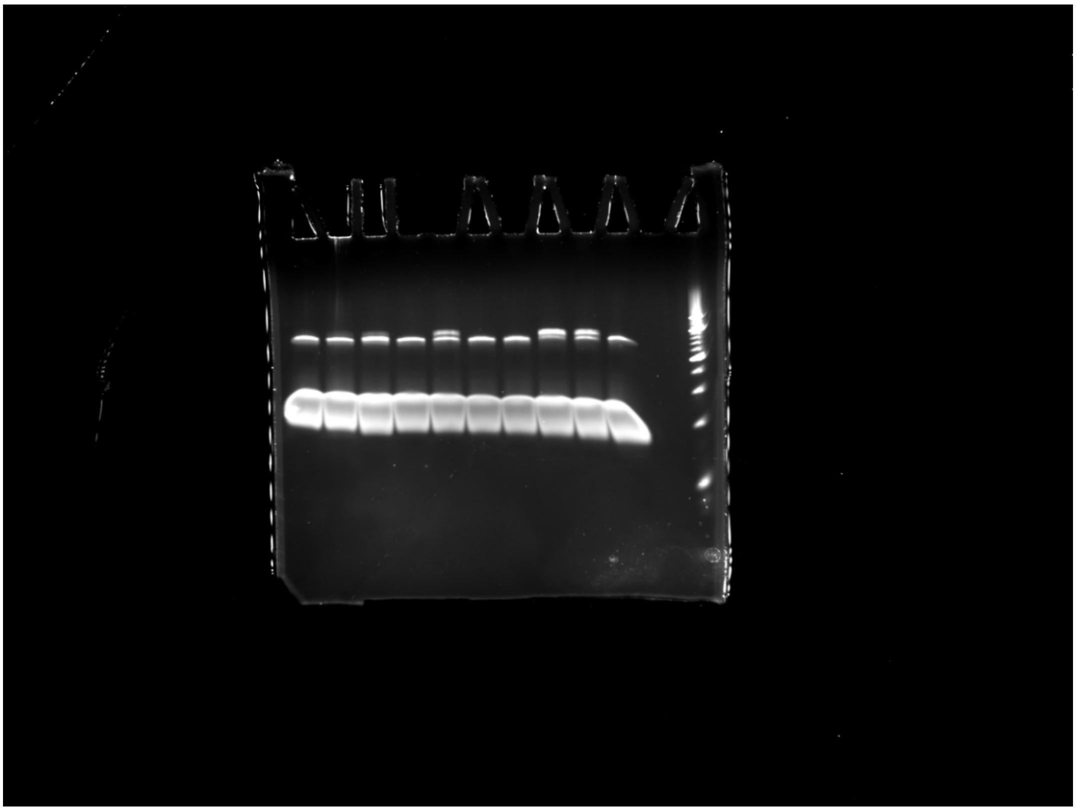

Figure 7C

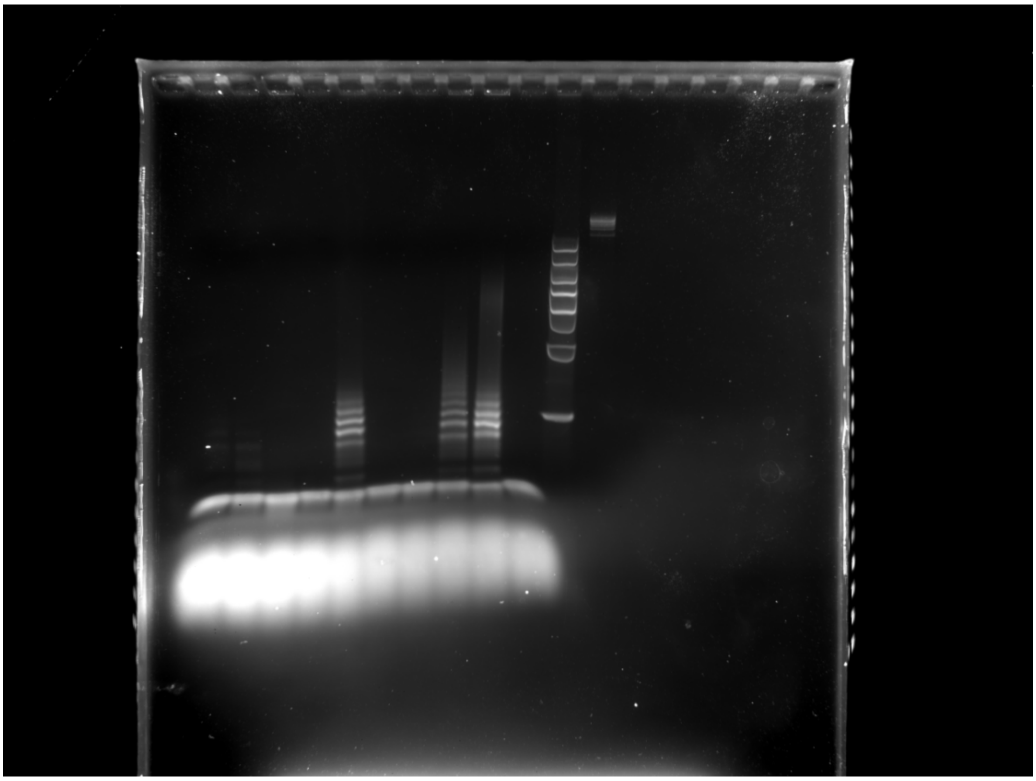

Figure 7D

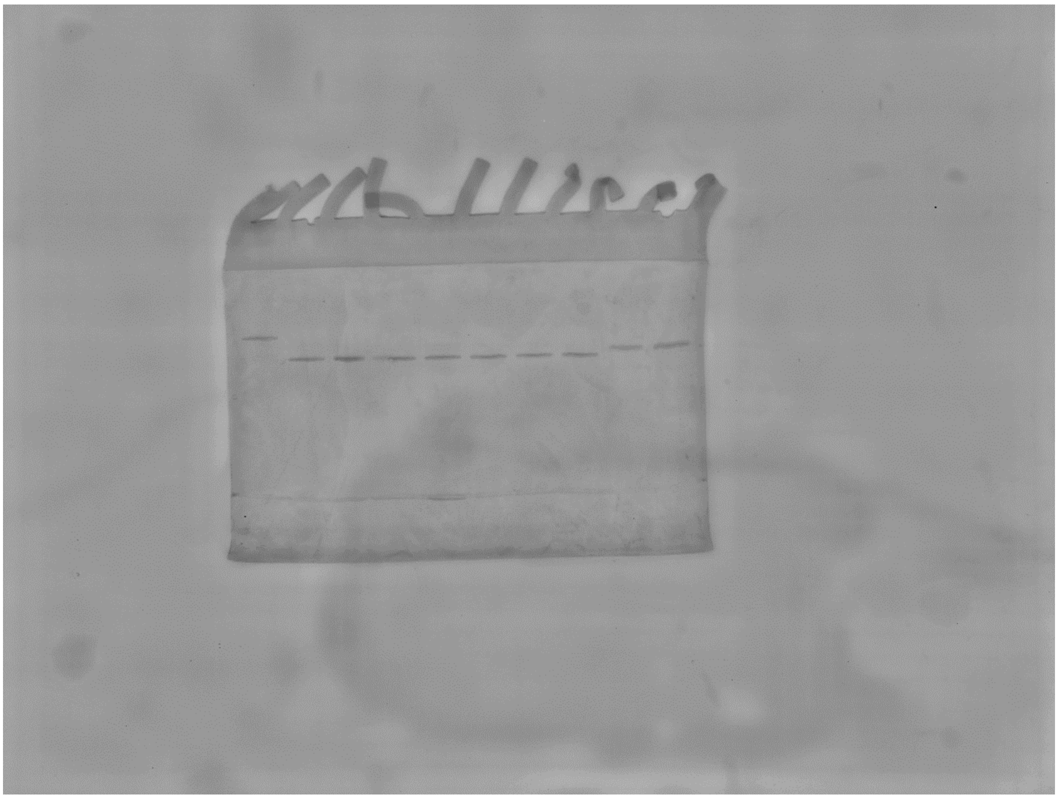

Supplementary Figure 1A

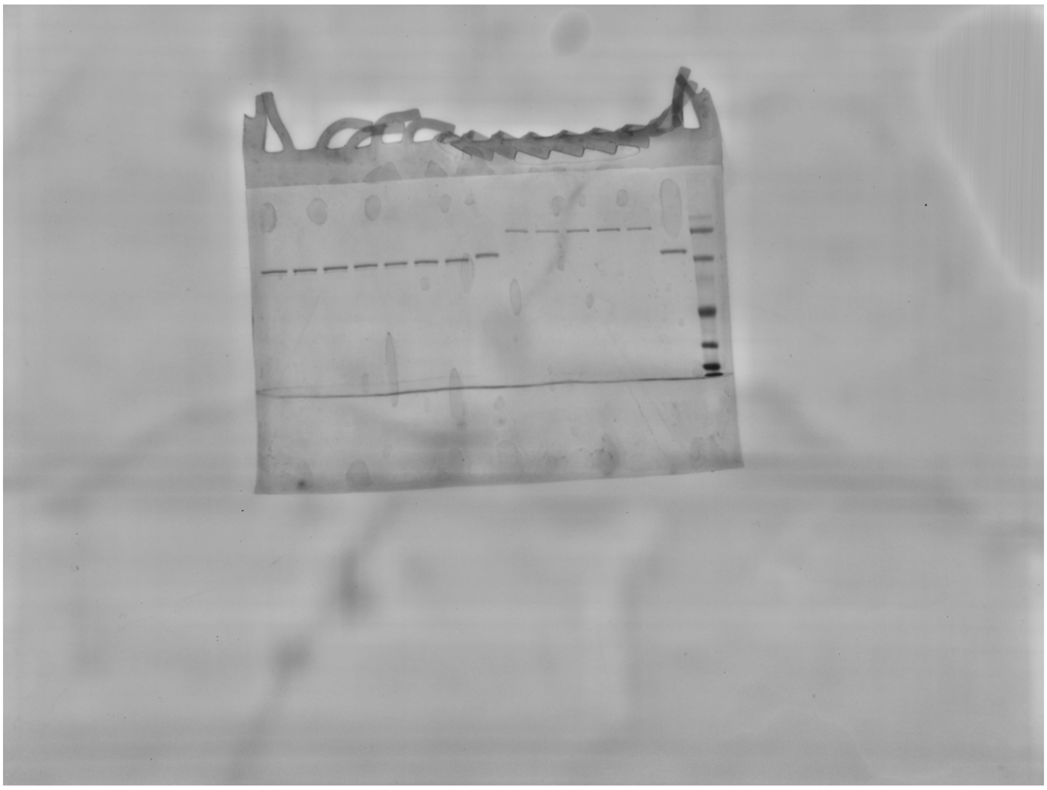

Supplementary Figure 1B

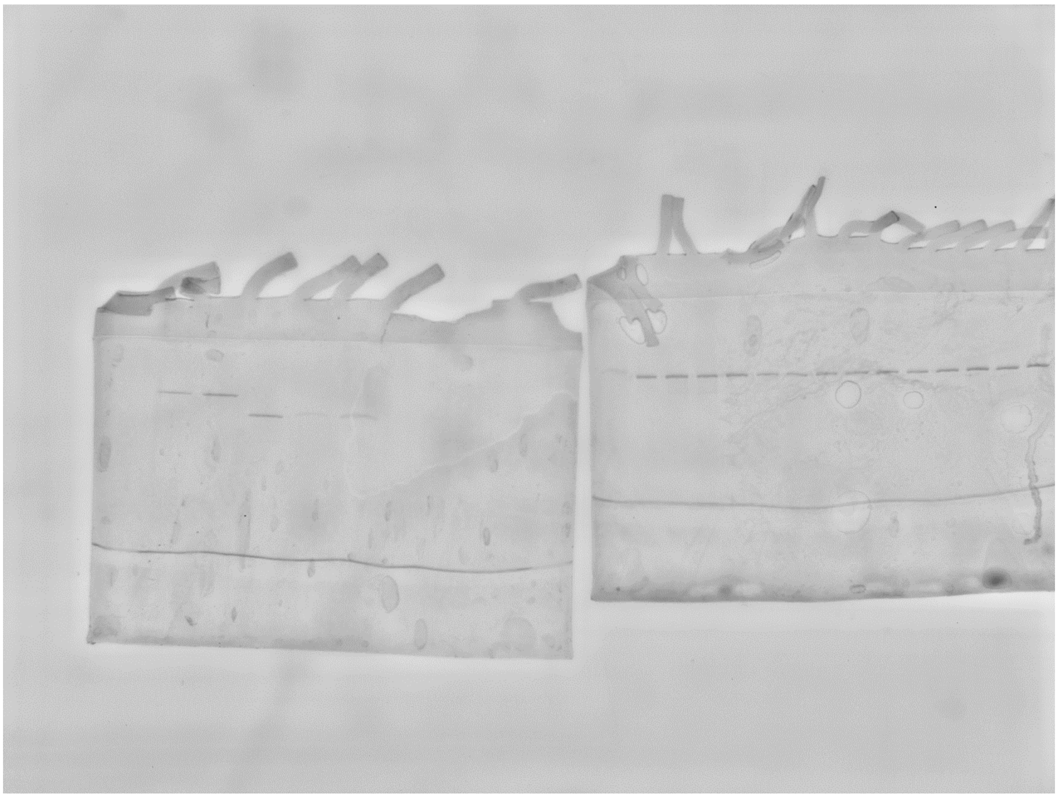

Supplementary Figure 1C

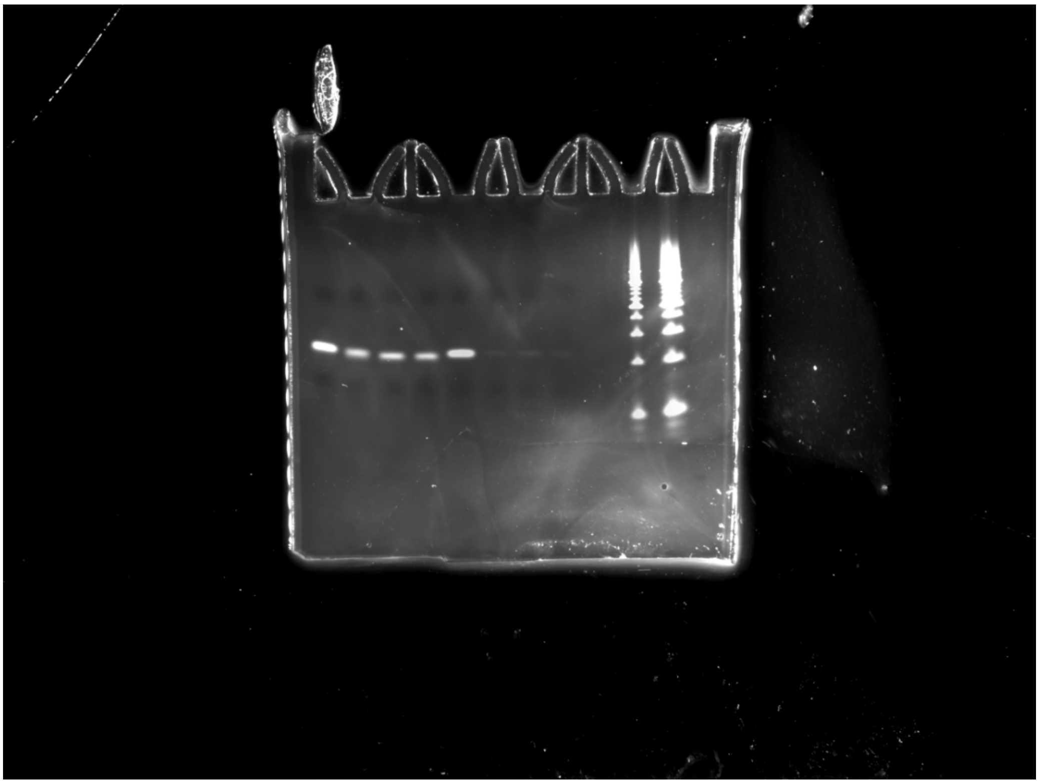

Supplementary Figure 2A

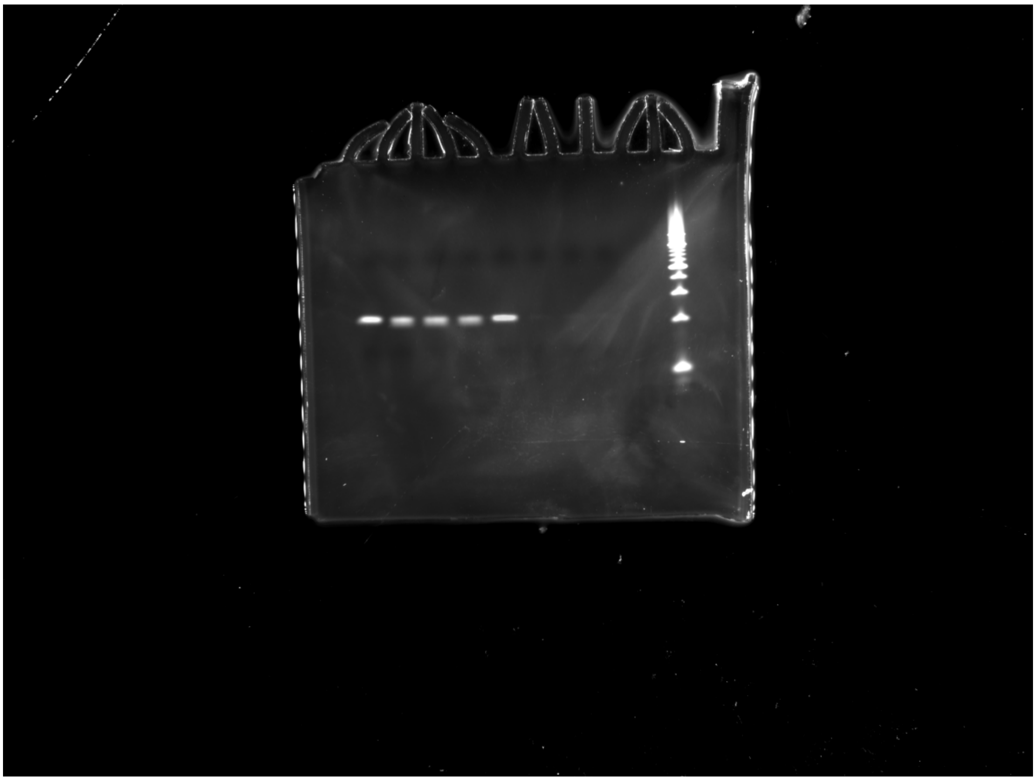

Supplementary Figure 2B

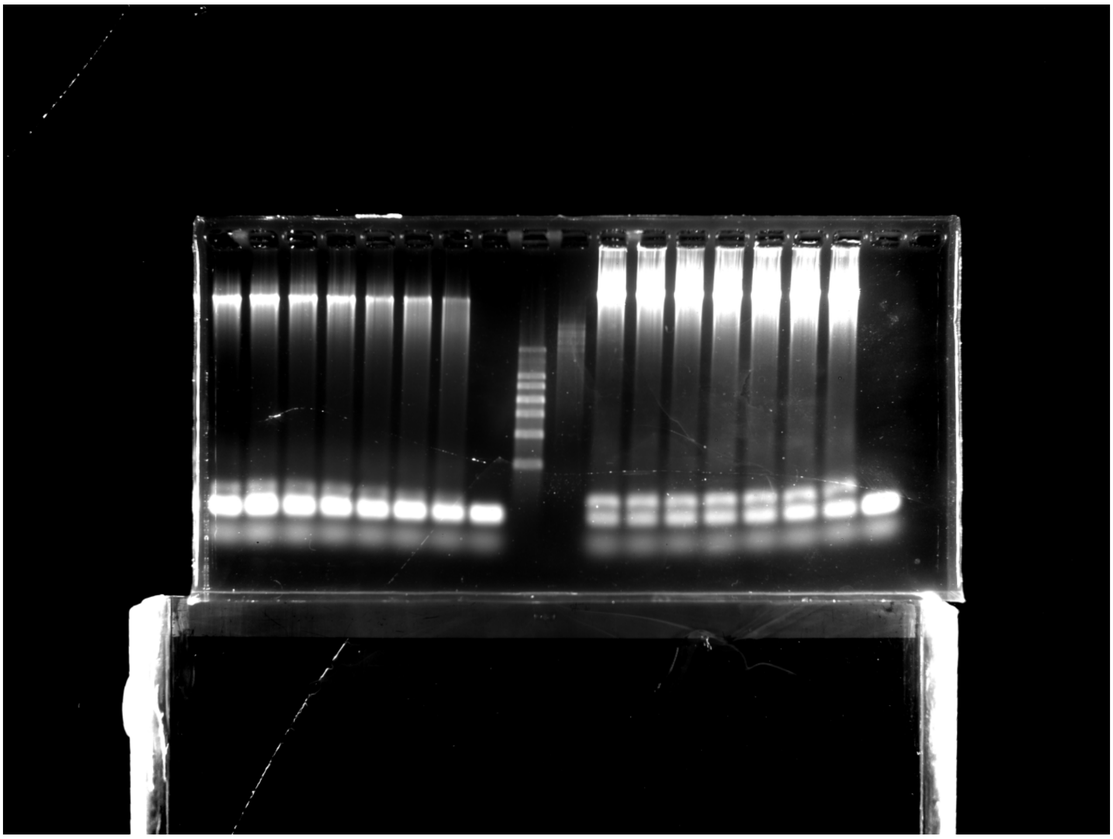

Supplementary Figure 3A

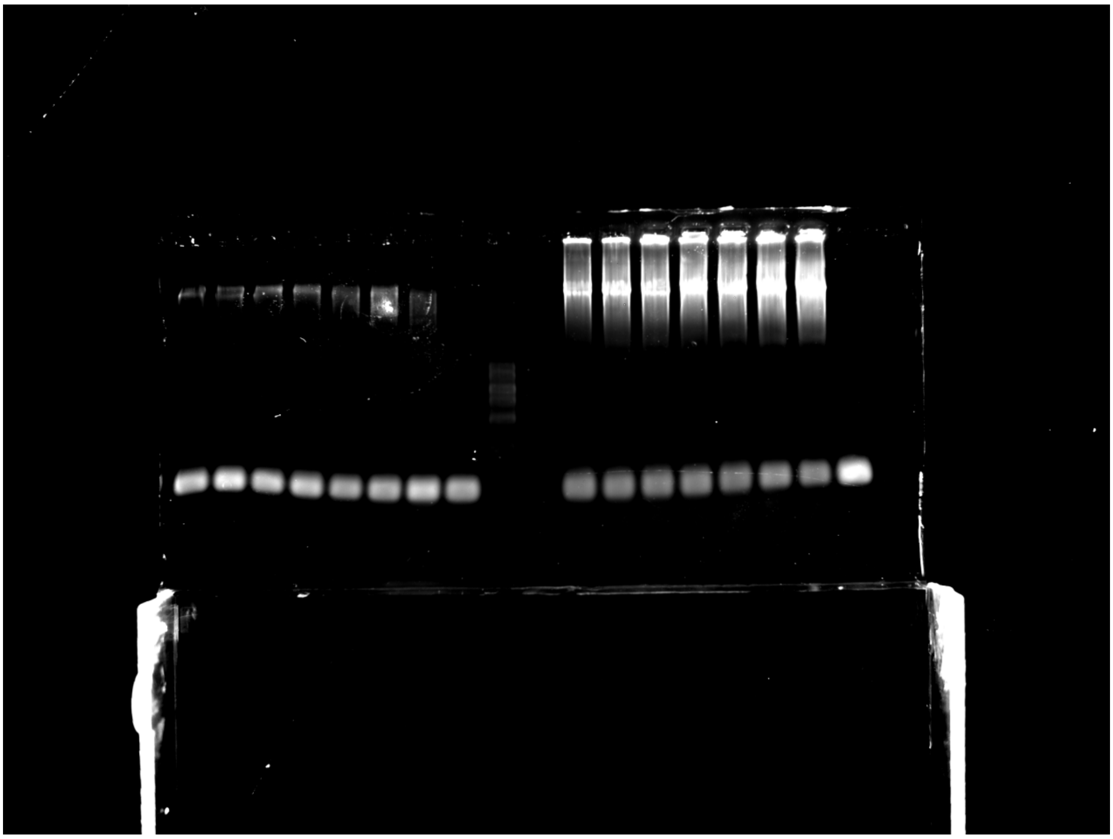

Supplementary Figure 3B

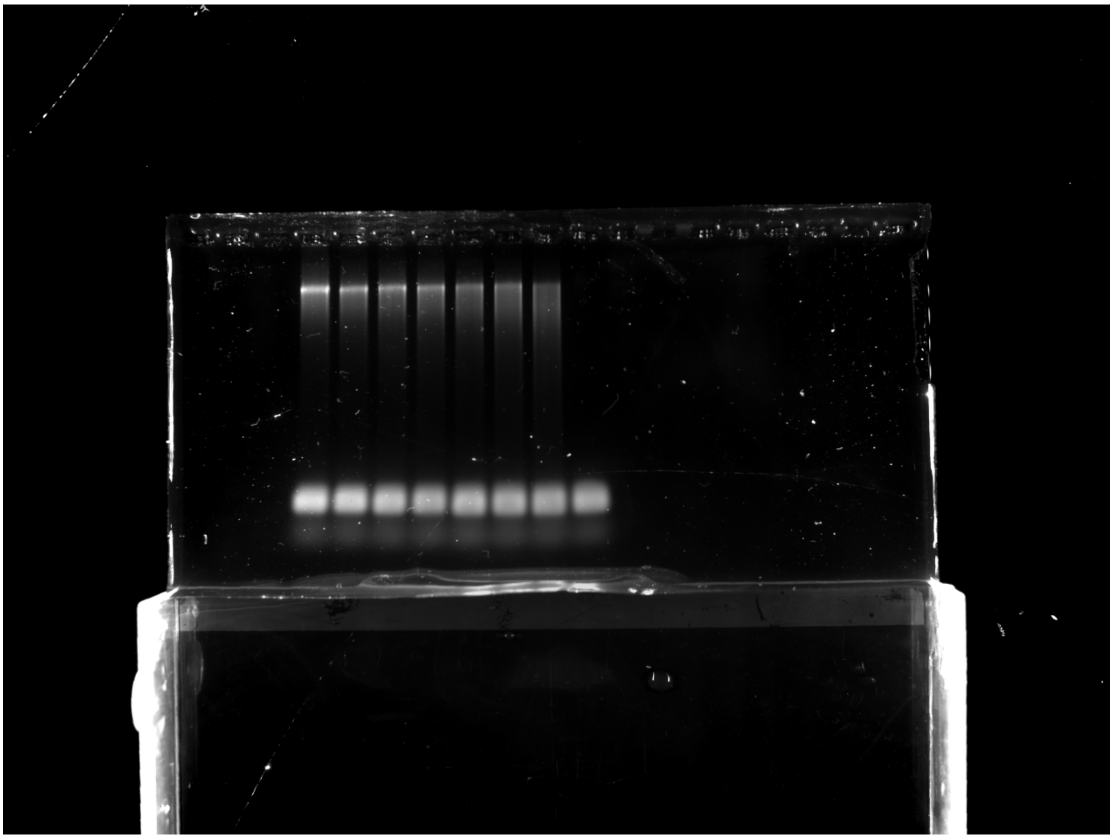

Supplementary Figure 3C

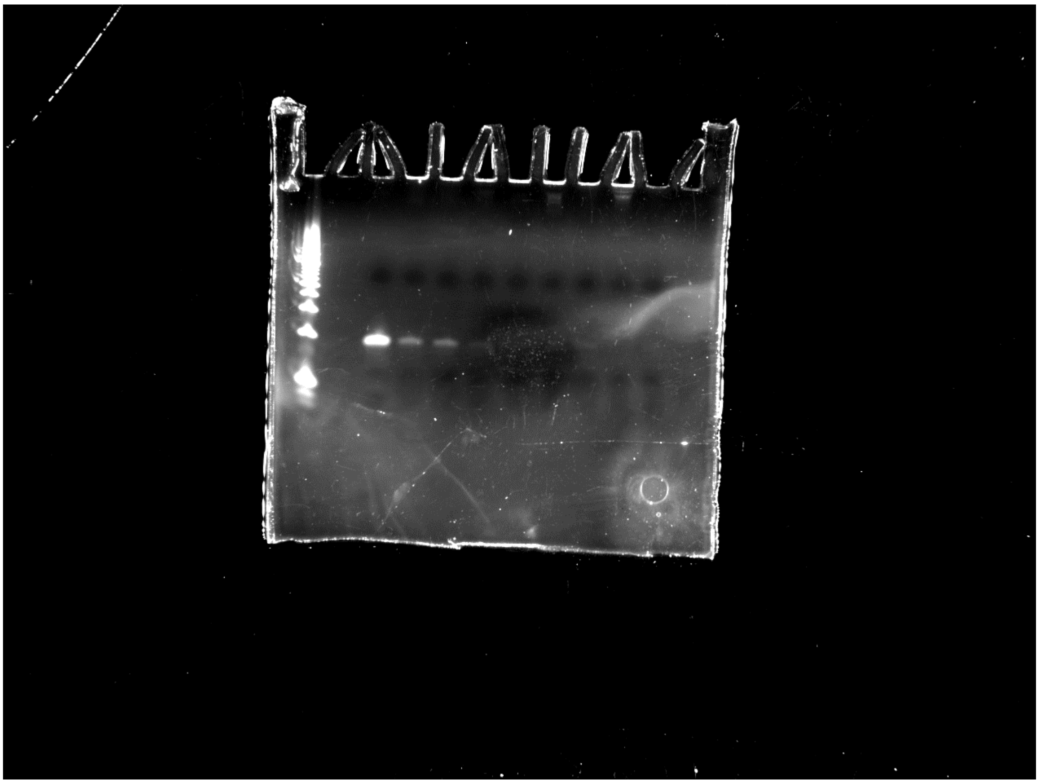

Supplementary Figure 7A

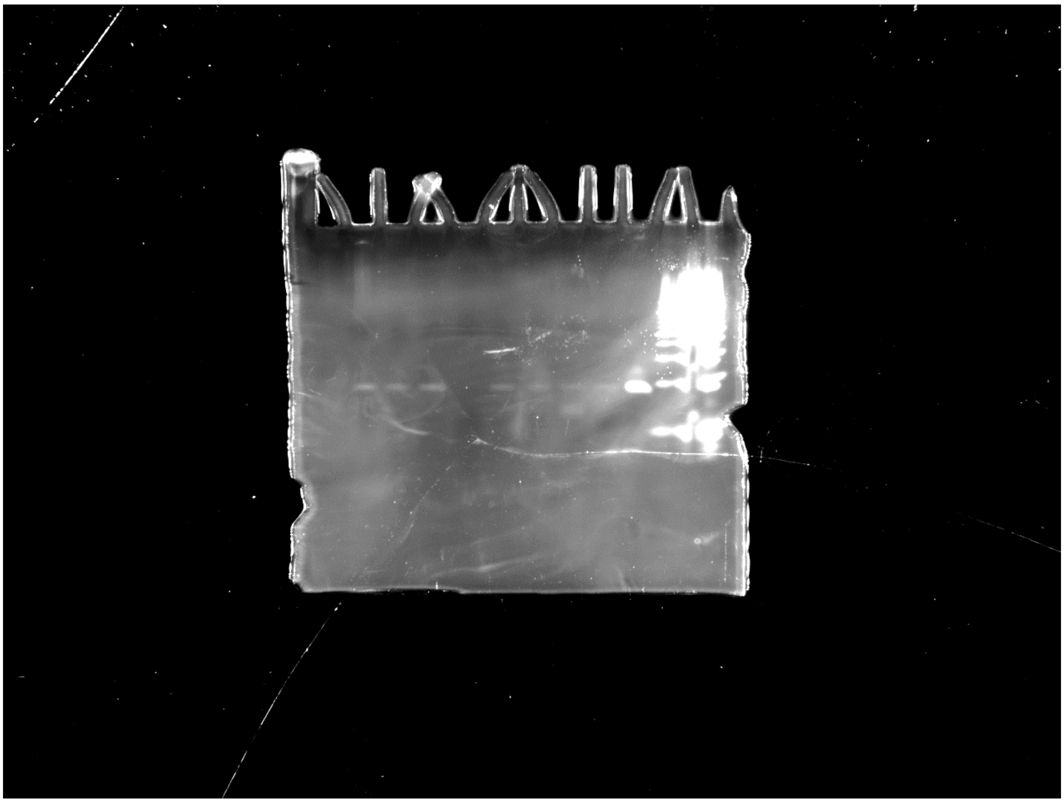

Supplementary Figure 7B

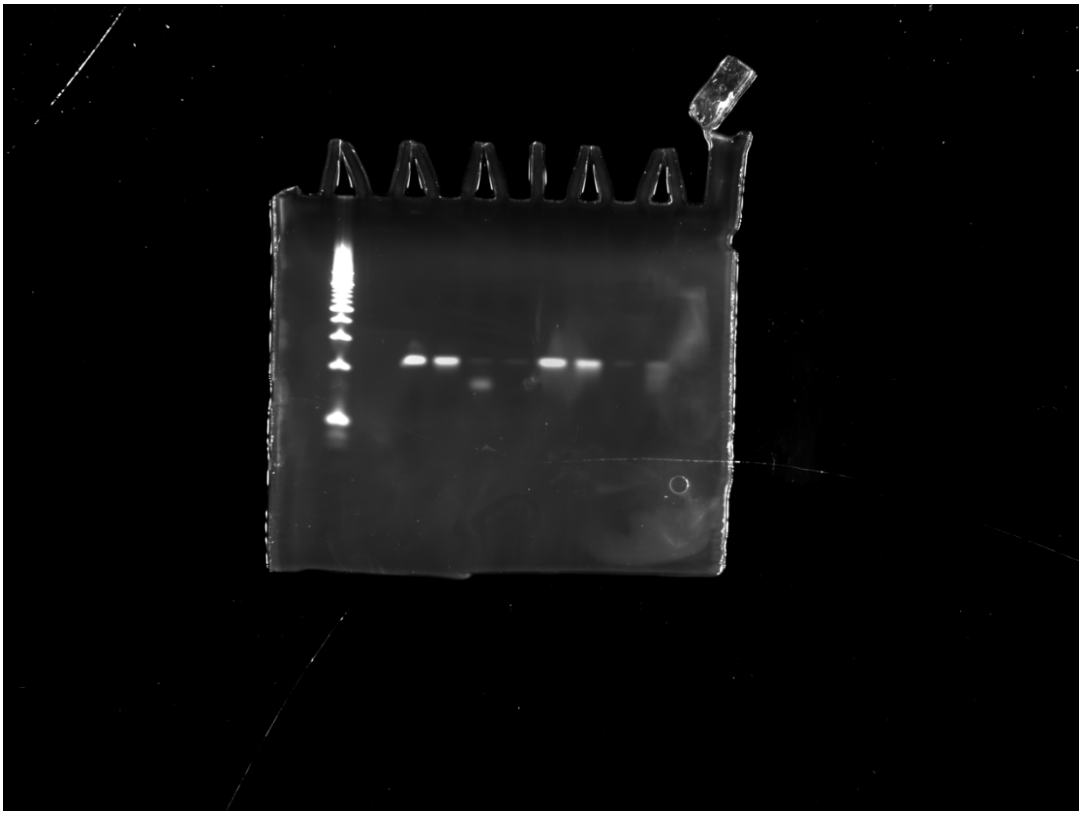

Supplementary Figure 7C
