# Supplementary material for: Characterization and Engineering of Two Novel Strand-Displacing B Family DNA Polymerases from Bacillus Phage SRT01hs and BeachBum
Source: Biomolecules. 2025 Aug 5;15(8):1126. doi: 10.3390/biom15081126 (PMC12383890; doi:10.3390/biom15081126)
Supplement: Supplementary file 1 [file biomolecules-15-01126-s001.zip › Supplementary Table S1.pdf]

Supplementary Table S1 DNA polymerases with strand displacement from Family B DNA polymerases

Name	3'-5'Exonuclease activity	Strand Displacement	Processivity	Temperature	Catalytic bivalent metal cations (dNTPs as substrates)
Phi29	Yes	Yes		10℃-45℃	Mg^{2+}, Mn^{2+}, Fe^{2+}
BBum	Yes	Yes	similar to Phi29	10℃-45℃	Mg^{2+}, Mn^{2+}, Fe^{2+}
SRHS	Yes	Yes	similar to Phi29	10℃-45℃	Mg^{2+}, Mn^{2+}, Fe^{2+}
IME199 [5]	Yes	Yes	similar to Phi29	15℃-35℃	Mg^{2+}, Mn^{2+}, Ca^{2+}
Bam35 [3]	Yes	Yes	Unknown (unreported)	37℃ (unreported range)	Mg^{2+} (unreported other tests)
