# Supplementary figures and images for: Characterization and Engineering of Two Novel Strand-Displacing B Family DNA Polymerases from Bacillus Phage SRT01hs and BeachBum
Source: Biomolecules. 2025 Aug 5;15(8):1126. doi: 10.3390/biom15081126 (PMC12383890; doi:10.3390/biom15081126)

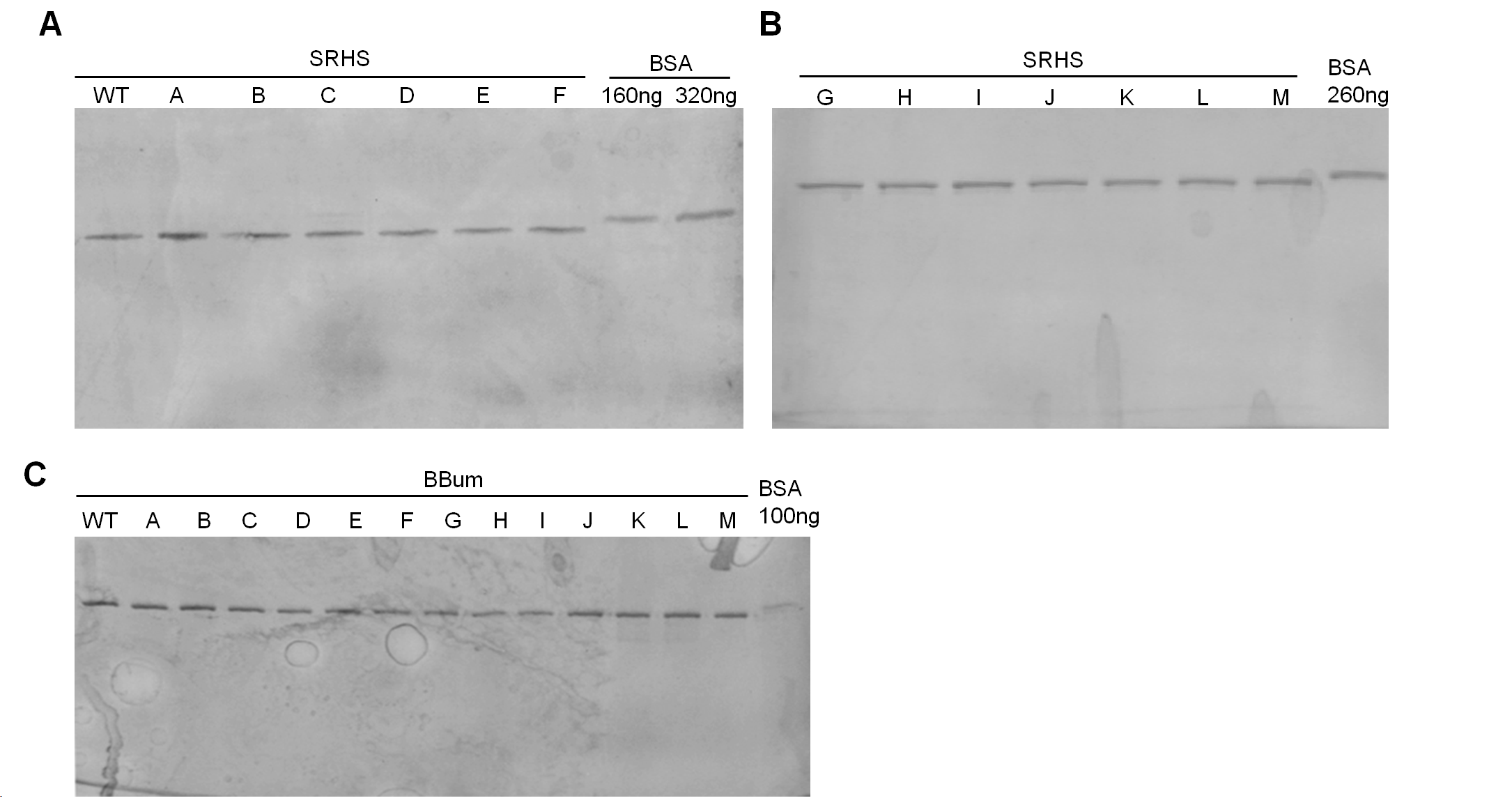

Supplement: Supplementary file 1 [file biomolecules-15-01126-s001.zip › Supplementary Figure S1.tif]

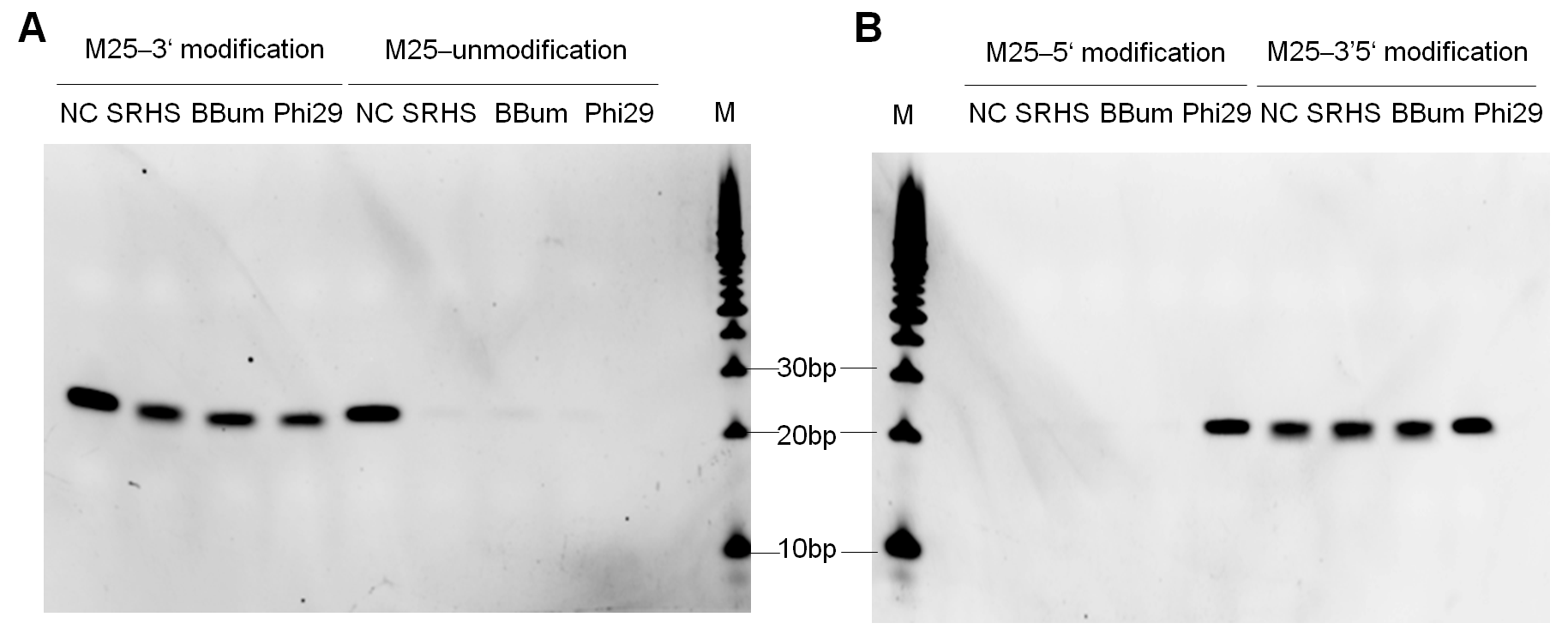

Supplement: Supplementary file 1 [file biomolecules-15-01126-s001.zip › Supplementary Figure S2.tif]

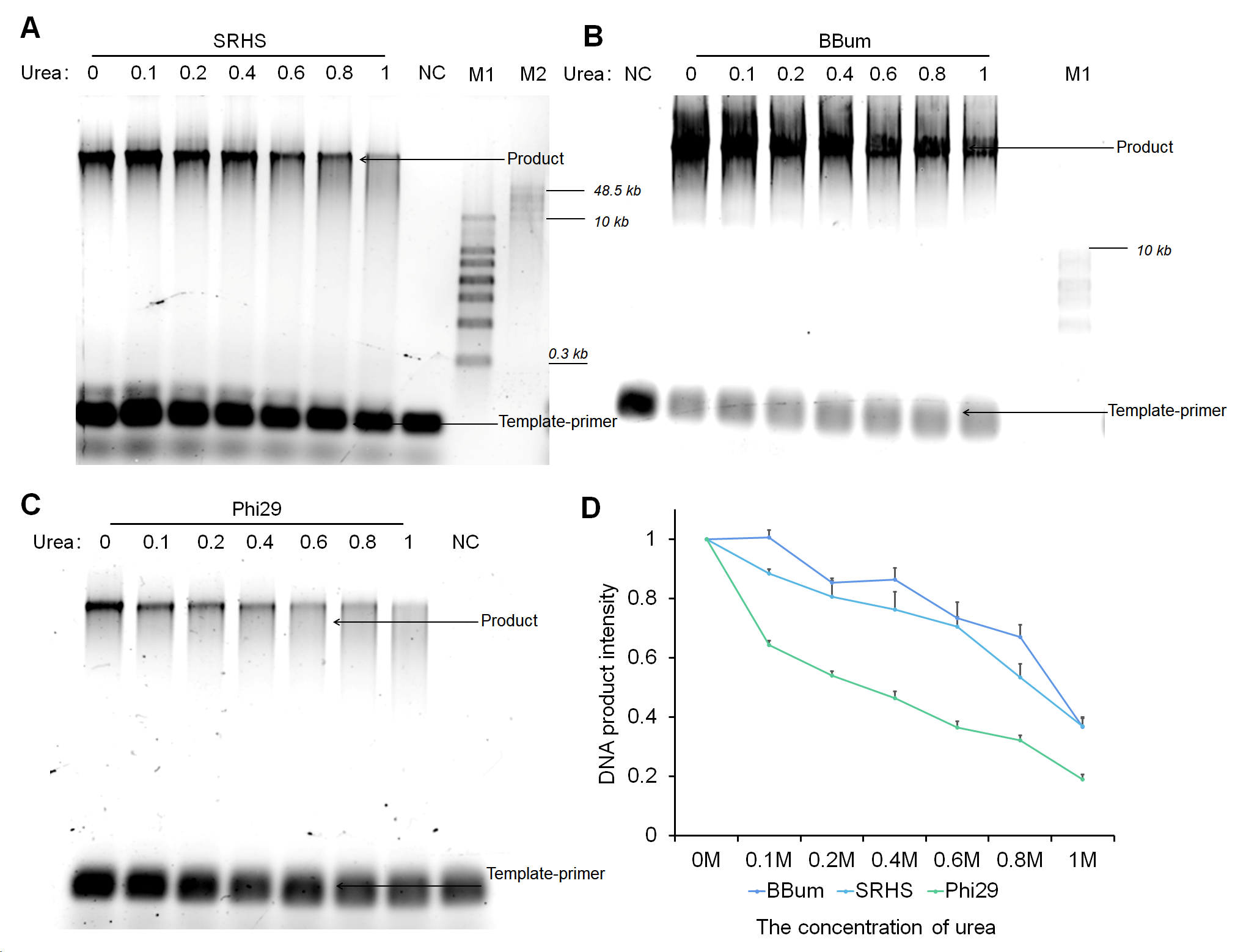

Supplement: Supplementary file 1 [file biomolecules-15-01126-s001.zip › Supplementary Figure S3.tif]

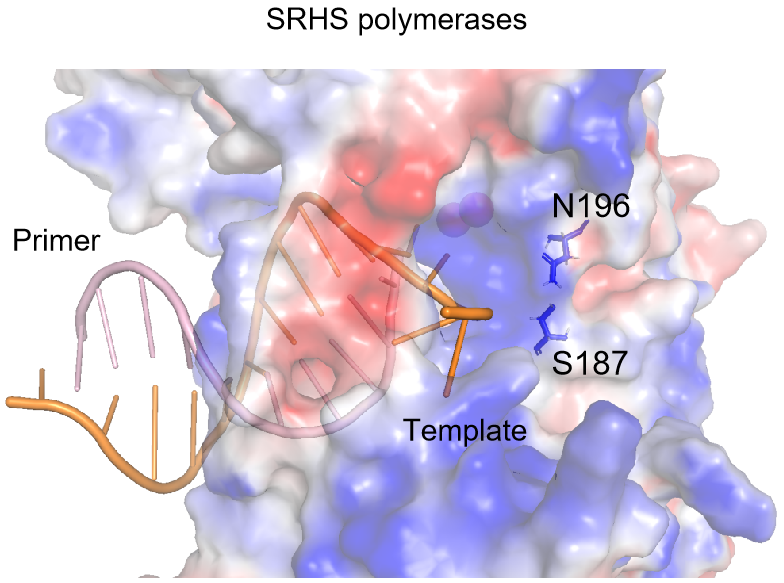

Supplement: Supplementary file 1 [file biomolecules-15-01126-s001.zip › Supplementary Figure S4.tif]

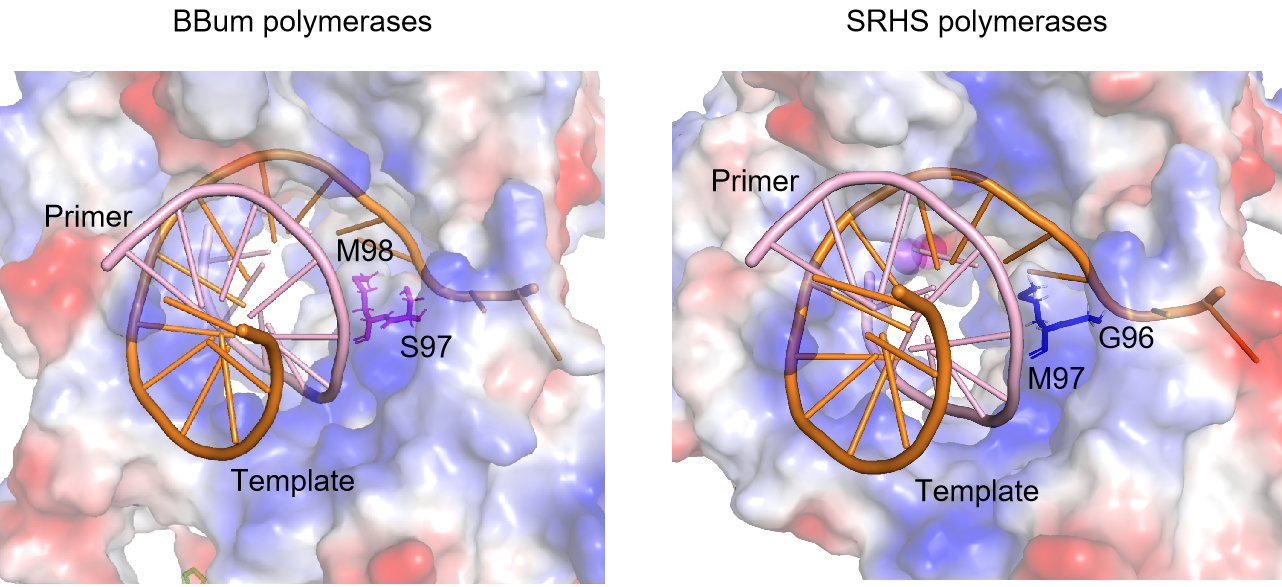

Supplement: Supplementary file 1 [file biomolecules-15-01126-s001.zip › Supplementary Figure S5.tif]

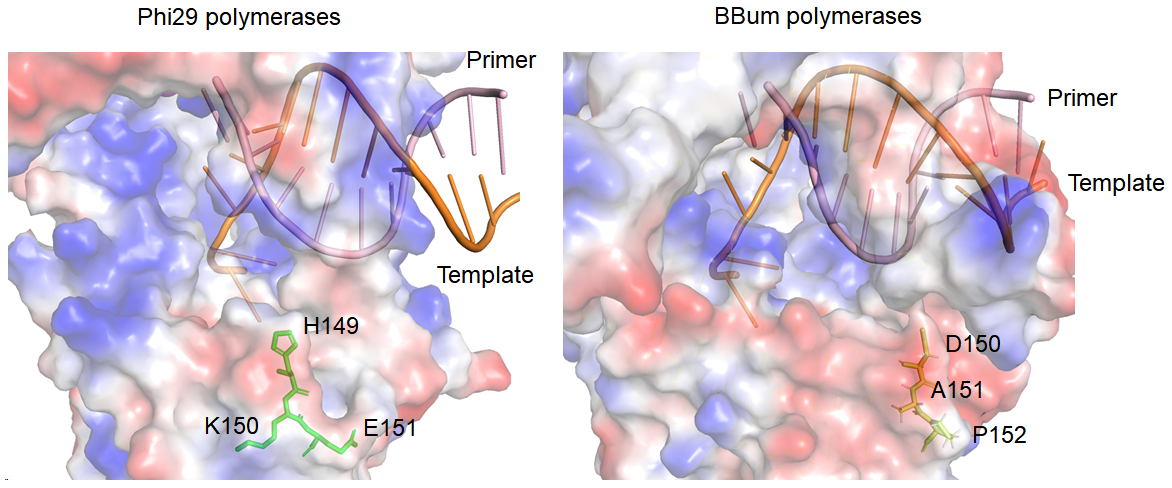

Supplement: Supplementary file 1 [file biomolecules-15-01126-s001.zip › Supplementary Figure S6.tif]

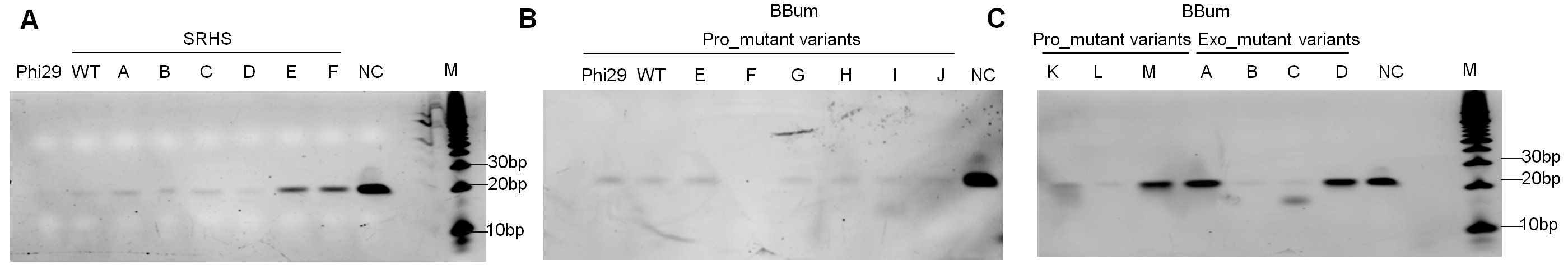

Supplement: Supplementary file 1 [file biomolecules-15-01126-s001.zip › Supplementary Figure S7.tif]

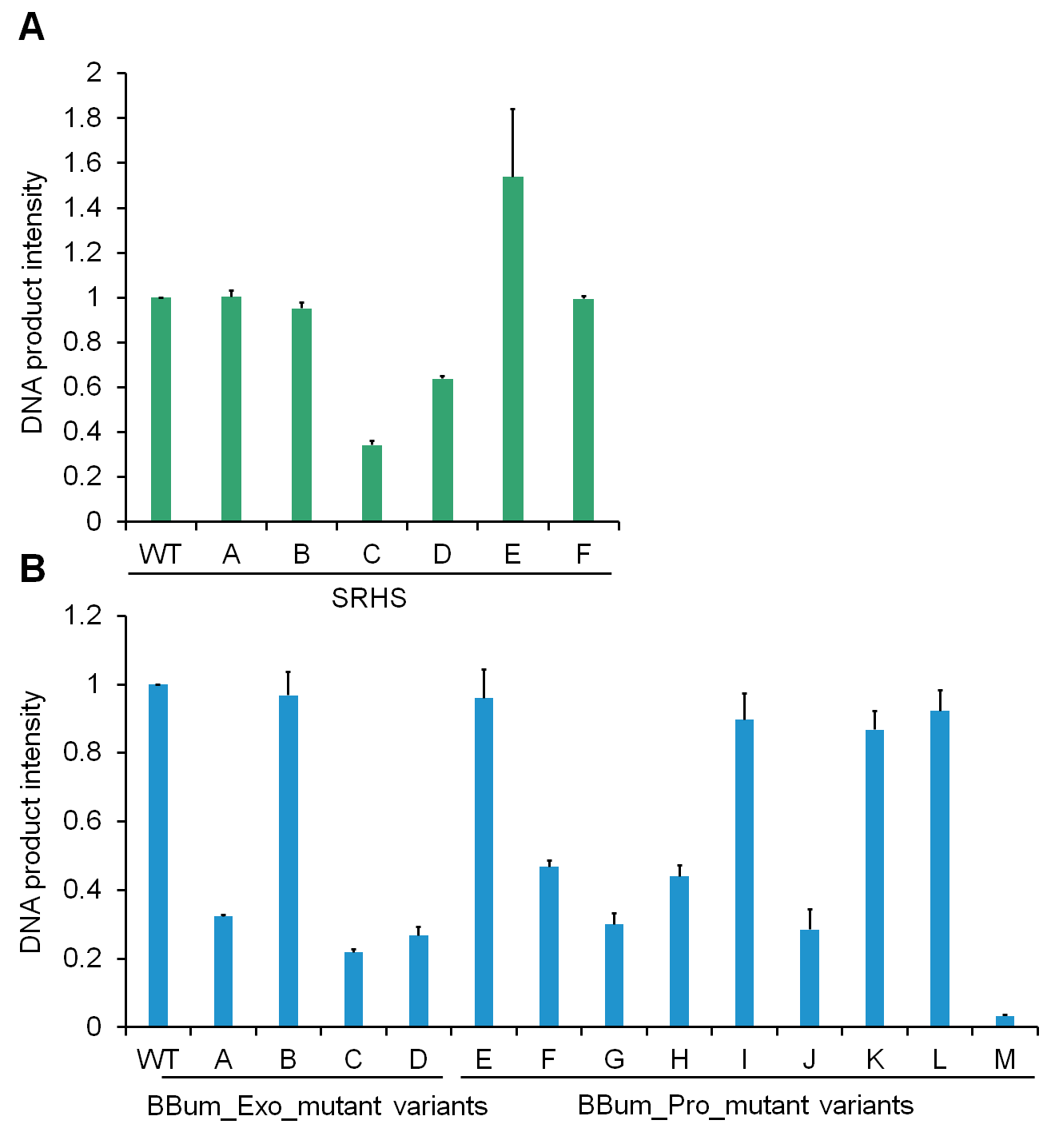

Supplement: Supplementary file 1 [file biomolecules-15-01126-s001.zip › Supplementary Figure S8.tif]
